# Polymerisation‐Induced Self‐Assembly of Graft Copolymers

**DOI:** 10.1002/anie.202210518

**Published:** 2022-09-29

**Authors:** Satu Häkkinen, Joji Tanaka, Ramón Garcia Maset, Stephen C. L. Hall, Steven Huband, Julia Y. Rho, Qiao Song, Sébastien Perrier

**Affiliations:** ^1^ Department of Chemistry University of Warwick Coventry CV4 7AL UK; ^2^ Department of Chemistry University of North Carolina at Chapel Hill Chapel Hill NC 27599-3290 USA; ^3^ ISIS Neutron and Muon Source Rutherford Appleton Laboratory Didcot OX11 0QX UK; ^4^ Warwick Medical School University of Warwick Coventry CV4 7AL UK

**Keywords:** Graft Copolymer, PISA, Polymerization, RAFT, Self-Assembly

## Abstract

We report the polymerisation‐induced self‐assembly of poly(lauryl methacrylate)‐*graft*‐poly(benzyl methacrylate) copolymers during reversible addition‐fragmentation chain transfer (RAFT) *grafting from* polymerisation in a backbone‐selective solvent. Electron microscopy images suggest the phase separation of grafts to result in a network of spherical particles, due to the ability of the branched architecture to freeze chain entanglements and to bridge core domains. Small‐angle X‐ray scattering data suggest the architecture promotes the formation of multicore micelles, the core morphology of which transitions from spheres to worms, vesicles, and inverted micelles with increasing volume fraction of the grafts. A time‐resolved SAXS study is presented to illustrate the formation of the inverted phase during a polymerisation. The grafted architecture gives access to unusual morphologies and provides exciting new handles for controlling the polymer structure and material properties.

## Graphical abstract

The influence of branching on polymer self‐assembly is investigated by conducting polymerisation‐induced self‐assembly of sparsely grafted poly(lauryl methacrylate)‐*graft*‐poly(benzyl methacrylate) in *n*‐dodecane. Electron microscopy and X‐ray scattering techniques are used to characterise the resulting materials.

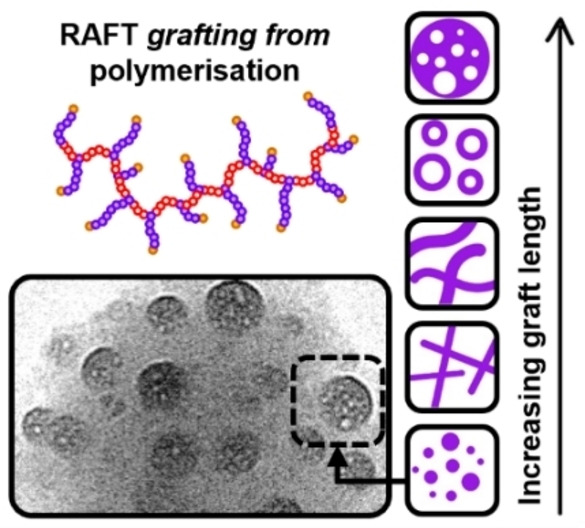

## Introduction

The field of polymer self‐assembly has allowed the development of functional materials with wide ranging uses such as adhesives,^[1]^ energy storage,^[2]^ membranes,^[3]^ lubrication,^[4]^ and bioapplications.[^5^, ^6^] Prospective applications continue to drive further research in this area.[^7^, ^8^, ^9^, ^10^] Amongst the parameters that dictate the outcome of polymer self‐assembly,[^11^, ^12^] polymer architecture has been found to play an important role and may be used to direct aggregation pathways.[^13^, ^14^, ^15^, ^16^, ^17^] The simplest and most studied polymer architecture to date is the linear AB diblock copolymer, the self‐assembly of which is well understood in bulk and solution. In selective solvents, the morphology of diblock copolymer aggregates typically evolves from spherical micelles to cylindrical micelles, bilayers, and inverted structures such as large compound micelles with increasing volume fraction of the solvophobic block.^[11]^ A third block may be added to access morphologies such as multicompartment micelles with chemically distinct core domains.^[13]^ The introduction of branching points to the polymer structure can influence aggregation by causing solvophilic segments to form loops near the separation interface, or to bridge adjacent core domains.^[18]^

Solution self‐assembly may be carried out in various ways, such as by using the conventional co‐solvent or film rehydration methods.^[11]^ Research involving controlled radical polymerisation has given rise to an alternative approach wherein aggregation takes place during polymerisation in a selective solvent.[^19^, ^20^] This polymerisation‐induced self‐assembly (PISA) strategy uses the increasing solvophobicity of a propagating polymer chain to induce its self‐assembly in situ, and may be used to prepare nanoparticles of various morphologies and chemistries in polar or non‐polar media.[^21^, ^22^] Reactions can be conducted at high concentrations without the need for post‐polymerisation protocols. A typical reaction involves the chain‐extension of a soluble polymer with a second monomer in a dispersion or an emulsion polymerisation. The convenience and versatility of PISA is manifested in the fast‐growing number of publications since its discovery, and the many applications proposed for the materials.[^4^, ^5^, ^23^, ^24^]

In recent years, the use of complex polymer architectures for PISA has sparked some interest.^[25]^ Pioneering studies have revealed new possibilities using triblock[^26^, ^27^, ^28^, ^29^, ^30^] and star‐like architectures.[^31^, ^32^, ^33^, ^34^] For instance, Armes and co‐workers found diblock copolymer vesicles to transition into framboidal vesicles upon chain‐extension into a double solvophobic ABC triblock terpolymer.^[29]^ The surface roughness of the vesicles could be controlled by varying the length of the C‐block. Double solvophobic BAB triblock copolymers have been reported to form connected particles as a result of phase separation of B‐blocks of one molecule into separate core domains.[^28^, ^30^, ^35^] These examples amongst others suggest that exploring PISA beyond the classical diblock copolymers will broaden the characteristics and the scope of the materials prepared in this way.

Moving towards grafted architectures, some research has been done at the boundary of linear and grafted polymers by using oligomeric ethylene glycol^[36]^ or dimethyl siloxane^[37]^ macromonomers and monomers with large alkyl side groups such as lauryl^[38]^ or stearyl^[39]^ methacrylates. For example, Torres‐Rocha et al. used a polynorbornene‐*graft*‐poly(ethylene glycol) block to stabilise poly(cyclooctadiene) in water, resulting in a brush‐coil type architecture.^[40]^ Early examples of PISA during graft polymerisation involved alginate^[54]^ and dextran^[55]^ macroinitiators, and lead to spherical, worm‐like, or vesicular morphologies. During the course of our study, the PISA of poly(vinyl alcohol)‐*graft*‐poly(2‐hydroxypropyl methacrylate) and poly(vinyl alcohol)‐*graft*‐poly(diacetone acrylamide) copolymers were reported, for which only spherical micelles were found.^[41]^ Ferji and coworkers very recently reported the evolution of multicompartment vesicles and large compound nano‐objects during the PISA of dextran‐*graft*‐poly(2‐hydroxypropyl methacrylate) copolymers.^[56]^

We hypothesised that PISA of graft copolymers should reveal differences to their linear diblock analogues due to the influence of branching on core chain packing. To this end, the dispersion polymerisation of sparsely grafted (*n*_g_<10 %) poly(lauryl methacrylate)‐*graft*‐poly(benzyl methacrylate) (pLMA‐*g*‐pBzMA) copolymers was conducted in *n*‐dodecane using the RAFT *grafting from* approach.^[42]^ PISA of linear poly(lauryl methacrylate)‐*block*‐poly(benzyl methacrylate) diblock copolymers has been studied in detail by Armes and co‐workers, and was found to yield spherical micelles, worm‐like micelles, and vesicles across a range of block ratios.[^38^, ^43^, ^44^] We examined the influence of backbone length, graft length, grafting density, and concentration on the outcome of these reactions. The materials were characterised using electron microscopy (EM) techniques and small‐angle X‐ray scattering (SAXS). A time‐resolved SAXS experiment was used to observe self‐assembly take place in situ during a polymerisation to better understand the evolution of phase‐inverted morphologies.

## Results and Discussion

**Scheme 1:**
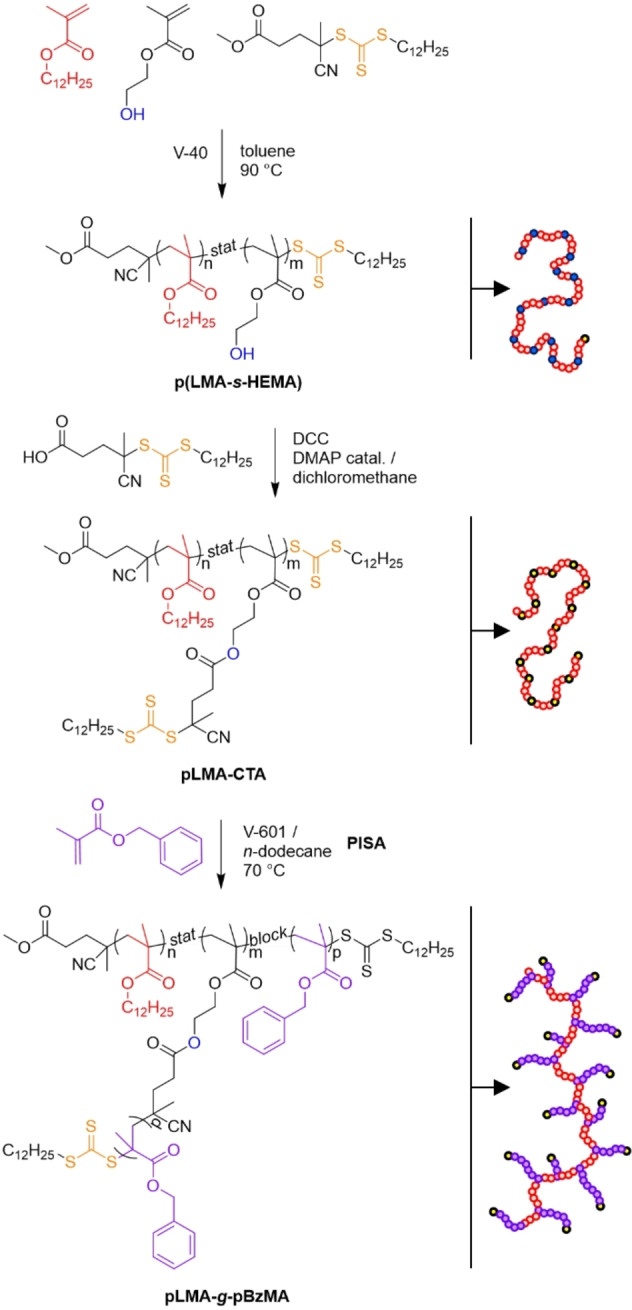
Synthetic route used in this study: RAFT copolymerisation of LMA and HEMA, post‐modification, and dispersion polymerisation of BzMA.

A previously reported copolymer/solvent system of solvophobic pBzMA and solvophilic pLMA in *n*‐dodecane was adapted for this study.^[38]^ In our design, solvophilic pLMA constituted the graft copolymer backbone, whereas pBzMA grafts became gradually solvophobic during their polymerisation. A backbone with pendent thiocarbonylthio grafting sites was constructed using a two‐step synthetic protocol, followed by dispersion polymerisation of BzMA to induce self‐assembly (Scheme 1).

### Preparation of Backbone Copolymers

**Figure 1:**
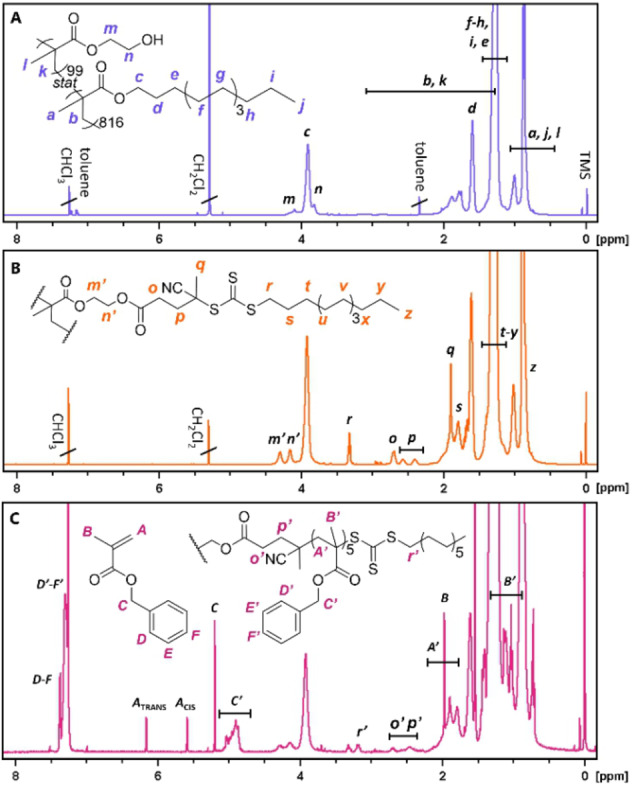
Representative ^1^H NMR spectra of polymers prepared in this study (400 MHz, CDCl_3_). A) Linear p(LMA_816_‐*s*‐HEMA_99_) (**1 a**). B) Functionalised backbone pLMA_915_‐CTA_10%_ (**1 b**). C) Reaction mixture after PISA with pLMA_915_‐CTA_10%_ targeting graft DP 5 (**6.5**).

**Figure 2:**
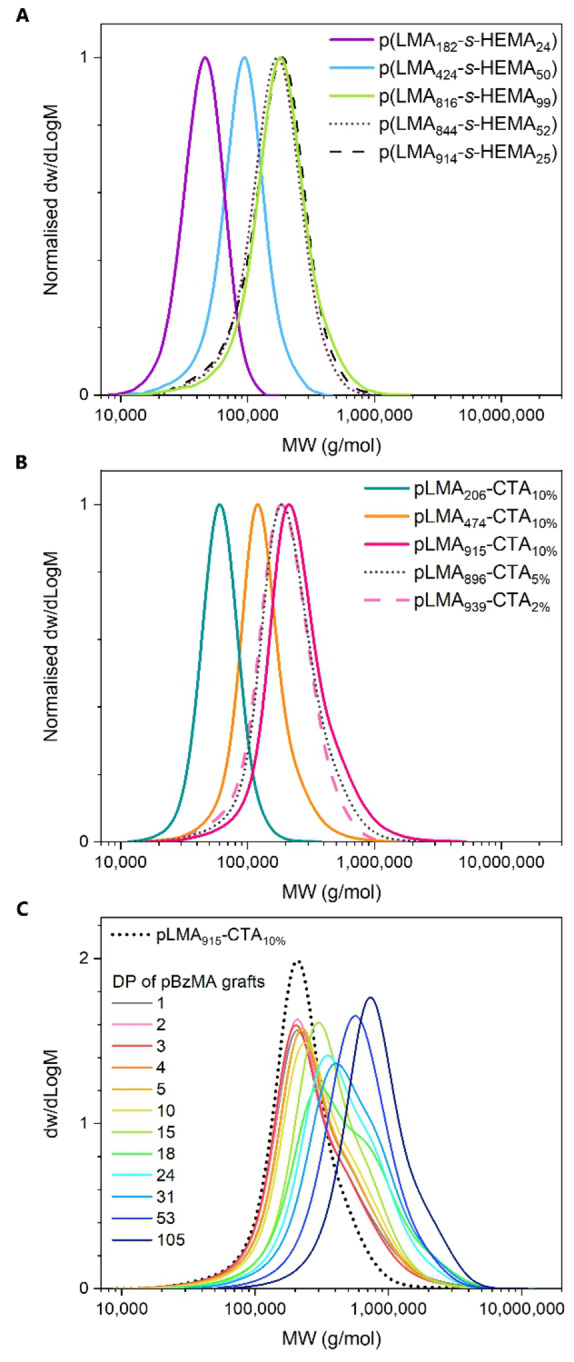
SEC profiles of A) p(LMA‐*s*‐HEMA) copolymers **1 a**–**5 a**, B) pLMA‐CTAs **1 b**–**5 b**, and C) pLMA‐*g*‐pBzMA graft copolymers **6.1**–**6.12** prepared with pLMA_915_‐CTA_10%_. Analysis in CHCl_3_ with DRI detection and PMMA calibration.

RAFT polymerisation of lauryl methacrylate (LMA) and 2‐hydroxyethyl methacrylate (HEMA) was used to prepare statistical poly[(lauryl methacrylate)‐*stat*‐(hydroxyethyl methacrylate)] (p(LMA‐*s*‐HEMA)) copolymers with controlled molecular weights (Table 1). HEMA was incorporated into the structure to introduce hydroxy groups, later used for functionalisation with 4‐cyano‐4‐(((dodecylthio)carbonothioyl)thio)pentanoic acid (CPADTC). The theoretical maximum number of branching points (*n*_CTA_) in each graft copolymer could be adjusted in this step with comonomer stoichiometry. Three copolymers with dissimilar degrees of polymerisation (DPs) of 206, 474, and 915 but similar comonomer ratios (11–12 mol% HEMA) were prepared to study the influence of backbone length on PISA. Conversely, three copolymers with dissimilar comonomer ratios of 2.7, 5.9, and 12 mol% HEMA but similar lengths (DP=896–939) were prepared to study the effect of grafting density. Monomer conversions were determined by ^1^H NMR in CDCl_3_ and **1 a** was further isolated to collect representative ^1^H NMR and ^1^H‐^13^C HSQC spectra to resolve overlapping signals (Figures 1A and S3–4). SEC analysis of **1 a**–**5 a** showed unimodal molecular weight distributions and reasonable dispersities for all polymers (Figure 2A). Polymers **1 a**–**3 a** exhibited large differences in elution times, indicating a significant difference in length. A good overlap was observed for **1 a**, **4 a**, and **5 a**, confirming these backbones were similar in length.

**Table 1:** Structural and characterisation details of backbone copolymers before and after functionalisation.

Statistical copolymers								Functionalised copolymers					
Structure^[a]^	Conv. [%]	*t* [h]	DP	*n*_HEMA_^[a]^ [mol %]	*M*_n,th_ [g mol^−1^]	*M*_n,SEC_^[b]^ [g mol^−1^]	*Đ* ^[b]^	Structure	*n*_CTA/_ *n*_LMA_^[c]^ [%]	*n*_CTA_^[d]^	*M*_n,th_ [g mol^−1^]	*M*_n,SEC_^[b]^ [g mol^−1^]	*Đ* ^[b]^
	LMA, HEMA												
**1 a** p(LMA_816_‐*s*‐HEMA_99_)	88, 96	26	915	11	221 000	152 000	1.38	**1 b** pLMA_915_‐CTA_10%_	10	82	254 000	207 000	1.48
**2 a** p(LMA_424_‐*s*‐HEMA_50_)	90, 97	22	474	11	115 000	80 400	1.24	**2 b** pLMA_474_‐CTA_10%_	10	46	133 000	114 000	1.28
**3 a** p(LMA_182_‐*s*‐HEMA_24_)	76, 94	22	206	12	49 800	40 700	1.16	**3 b** pLMA_206_‐CTA_10%_	10	19	57 200	55 900	1.16
**4 a** p(LMA_844_‐*s*‐HEMA_52_)	84, 99	14	896	6	222 000	140 000	1.32	**4 b** pLMA_896_‐CTA_5%_	5	36	236 000	175 000	1.41
**5 a** p(LMA_914_‐*s*‐HEMA_25_)	88, 98	15	939	3	236 000	143 000	1.37	**5 b** pLMA_939_‐CTA_2%_	2	17	243 000	162 000	1.36

[a] Calculated from conversion. [b] SEC analysis in CHCl_3_ with DRI detection and PMMA calibration. [c] Theoretical maximum grafting density, quantified with ^1^H NMR in CDCl_3_. [d] Number of CTAs (i.e., grafting sites) per molecule.

Polymerisation kinetics of a reaction carried out under the same conditions as **1 a** showed a pseudo first‐order reaction during the first 6 h (Figure S1). A slightly higher polymerisation rate of HEMA implied some drift in the distribution of the two monomers and therefore grafts along each chain. SEC analysis showed an increasing *M*_n_ and a decreasing dispersity with increasing conversion, indicative of a controlled polymerisation.^[45]^

CPADTC was coupled onto the hydroxy groups of **1 a**–**5 a** using the Steglich esterification method with 4‐dimethylaminopyridine and dicyclohexylcarbodiimide,^[46]^ yielding CTA‐functionalised backbones (pLMA‐CTA) **1 b**–**5 b**. ^1^H NMR was used to quantify CTA to LMA ratio (*n*_CTA_/*n*_LMA_), descriptive of the theoretical maximum grafting density (Figure 1B and Eq. S3). The ratio corresponded well to the ratio of HEMA to LMA in the copolymer, suggesting near quantitative functionalisation. The data was supported by the absence of −CH_2_OH signal of HEMA in the ^1^H‐^13^C HSQC spectra (Figure S6). SEC profiles of the polymers showed a slight increase of *M*_n_ due to changes in molecular weight, solubility, and/or bulkiness of the side groups, and otherwise similar molecular weight distributions to their precursors (Figure 2B).

### Dispersion Polymerisation of BzMA

RAFT dispersion polymerisation of BzMA from pLMA‐CTA was conducted at 70 °C in *n‐*dodecane for 12 h with dimethyl 2,2′‐azobis(2‐methylpropionate) (V‐601, [CTA]_0_/[I]_0_=40) thermal initiator without stirring. The conversion of BzMA was determined with ^1^H NMR in CDCl_3_ (Figure 1C). Total solids content (i.e., *m*_pL*M*A‐CTA_+*m*_Bz*M*A_) of 20 wt% (wt/wt) was used in all reactions unless otherwise specified.

Owing to the design of the functionalised backbones, the polymerisation of pBzMA grafts took place via the R‐group *grafting from* mechanism in which propagating grafts remain covalently bound to the backbone.^[47]^ The strategy is prone to intermolecular graft‐graft coupling arising from termination via combination, particularly under high radical concentrations and monomer‐starved conditions.[^42^, ^48^] Coupling can be suppressed to some extent using a moderate initiator concentration and a slow radical flux.

SEC analysis of graft copolymers prepared using backbones **1 b**–**3 b** under identical reaction conditions showed the use of a long backbone to result in a more pronounced high molecular weight shoulder than using a short backbone (Figures 2C and S13). This may be explained by a long backbone having a higher probability of coupling per molecule, therefore affecting a larger fraction of the sample. Reactions targeting moderate graft lengths (DP 18–31) seemed to exhibit more pronounced coupling than those targeting short graft lengths (DP 1–15), despite the latter having a higher instantaneous radical concentration throughout the reaction. A possible explanation is that the self‐assembly may increase the probability of coupling by bringing propagating grafts into close proximity. Longer, more solvophobic grafts would be confined into phase‐separated globules earlier in the reaction, leading to increased coupling. This was supported by the SEC data of 10 wt% reactions showing high molecular shoulders despite the reduced backbone and initiator concentration (Figure S17).

Statistical variation in the graft polymerisation resulted in a considerable fraction of uninitiated side group CTAs to remain when targeting short graft lengths (Table 2, Figure S7). While expected for short grafts,^[49]^ ^1^H NMR analysis showed the presence of ∼15 % uninitiated CTA even after targeting grafts with DP of 46, with reinitiation efficiency (I_eff_) quickly plateauing at DP 15–25. The trend was apparent in all reactions regardless of backbone length, graft length or concentration and resulted in deviation from the targeted grafting density.

**Table 2:** Structural and characterisation details of pLMA_915_‐*g*‐(pBzMA_x_)_y_ graft copolymers prepared with pLMA_915_‐CTA_10%_ to study the effect of graft length on PISA. The apparent graft length and the apparent number of grafts are denoted by x and y, respectively.

	Structure	Conv. [%]	DP_*ρ*_^[a]^	I_eff_^[b]^ [%]	DP_app_^[b]^	*n*_g,%_^[b]^	*n*_BzMA_/*n*_LMA_	*M*_n,th_ [g mol^−1^]	*M*_n,SEC_^[c]^ [g mol^−1^]	*Đ* ^[c]^
**6.1**	pLMA‐*g*‐(pBzMA_3_)_25_	76	1	30	3	3	0.086	266 000	206 000	1.82
**6.2**	pLMA‐*g*‐(pBzMA_4_)_32_	79	2	45	4	4	0.17	279 000	203 000	1.71
**6.3**	pLMA‐*g*‐(pBzMA_5_)_39_	82	3	53	5	5	0.28	293 000	199 000	1.76
**6.4**	pLMA‐*g*‐(pBzMA_6_)_46_	84	4	57	6	5	0.36	305 000	218 000	1.76
**6.5**	pLMA‐*g*‐(pBzMA_8_)_48_	84	5	60	8	5	0.47	321 000	221 000	1.73
**6.6**	pLMA‐*g*‐(pBzMA_14_)_57_	91	10	69	14	6	1.0	394 000	236 000	1.72
**6.7**	pLMA‐*g*‐(pBzMA_21_)_63_	96	15	73	21	7	1.6	477 000	288 000	1.56
**6.8**	pLMA‐*g*‐(pBzMA_24_)_61_	99	18	74	24	7	1.8	518 000	315 000	1.95
**6.9**	pLMA‐*g*‐(pBzMA_31_)_61_	97	24	77	31	7	2.4	598 000	341 000	1.68
**6.10**	pLMA‐*g*‐(pBzMA_40_)_61_	99	31	78	40	7	3.1	696 000	383 000	1.72
**6.11**	pLMA‐*g*‐(pBzMA_53_)_61_	98	53	N/A	–	–	5.3	1 020 000	481 000	1.50
**6.12**	pLMA‐*g*‐(pBzMA_105_)_61_	99	105	N/A	–	–	11	1 770 000	661 000	1.48

[a] Number‐average degree of polymerisation calculated from conversion, assuming 100 % reinitiation of side chain CTAs. [b] Reinitiation efficiency of side chain CTAs (I_eff_), apparent degree of polymerisation (DP_app_), and apparent grafting density (*n*_g,%_) calculated from lognormal CDF fit (Supporting Information, 3.4). [c] SEC analysis in CHCl_3_ with DRI detection and PMMA calibration.

Polymerisations were repeated in toluene (a non‐selective solvent) to rule out effects of self‐assembly. A lognormal cumulative distribution function was fitted to data gathered across 44 PISA reactions to account for incomplete reinitiation by calculating apparent graft lengths (DP_app_) and apparent grafting densities (*n*_g,%_) (Figure S9, Eq. S9–10).

### Effect of the Grafted Architecture on PISA

**Figure 3:**
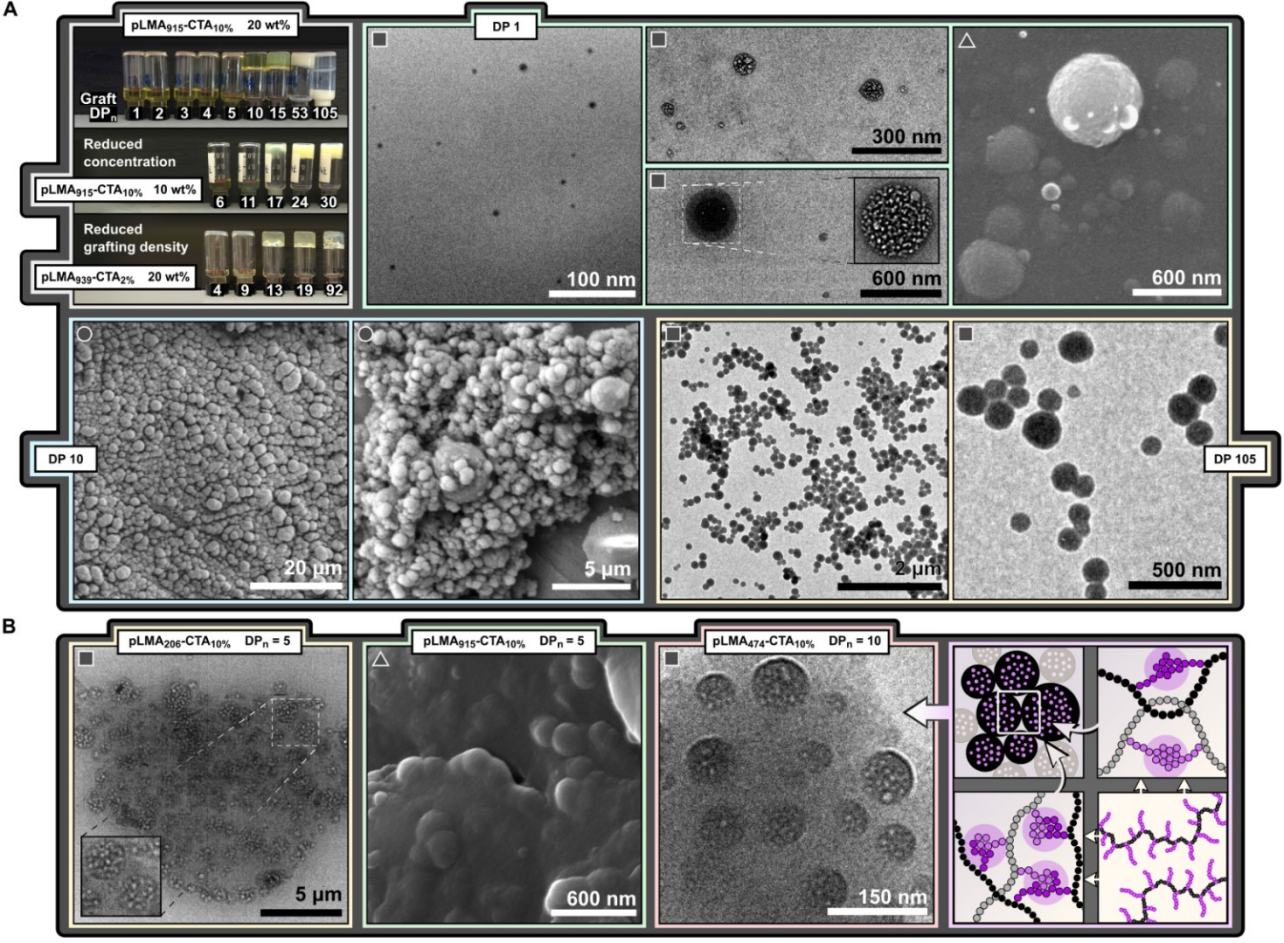
A) Reaction mixtures after PISA using pLMA_915_‐CTA_10%_ to target various graft lengths at 20 wt% and 10 wt% solids content, and using pLMA_939_‐CTA_2%_ to study the effects of grafting density. TEM (▪), SEM (▴), and cryo‐SEM (•) images show nanostructures prepared using pLMA_915_‐CTA_10%_ at 20 wt% targeting graft lengths of 1, 10, and 105 repeat units. B) Connected spheres obtained using pLMA_206_‐CTA_10%_, pLMA_915_‐CTA_10%_, and pLMA_474_‐CTA_10%_ at 20 wt%. Illustration shows the proposed origin of sphere clusters: phase separation of grafts leads to physical crosslinks arising from backbone entanglements and bridging of core domains.

A series of polymerisations was conducted with pLMA_915_‐CTA_10%_ **1 b** targeting a range of graft lengths (DP 1–105) (Table 2). The appearance of the reaction mixtures changed from dispersions with an increasing viscosity (DP≤5) into a gel‐like consistency (DP 10), and further into a low‐viscosity milky dispersion (DP 105) (Figure 3A). Within the gel‐like regime, an increase in turbidity (DP 15) was followed by a partial separation of solvent from the polymer phase at longer graft lengths (DP 50).

TEM, SEM, and cryo‐SEM imaging of the reaction mixtures revealed the presence of discrete spherical particles at the shortest and longest graft lengths (DP 1 and 105, respectively) and a connected/fused regime at moderate graft lengths (DP 10) (Figure 3A). The spheres formed at DP 1 were found to have an intricate internal structure and a rough surface with spherical protuberances. Detailed elucidation of the core morphology was unsuccessful due to destructive effects of the electron beam (Figure S19). Cryo‐SEM imaging of the gel‐like phase (DP 10) showed polydisperse spheres and clusters thereof. Despite appearing gel‐like at short time scales, the material could flow at long time scales (Figure S8).

Further reactions were carried out with backbones **2 b**–**5 b**. TEM of highly viscous reaction mixtures showed the presence of clustered spheres with a complex core structure (Figure 3B) The clusters were deposited to the substrate after attempted dilution of the reaction mixture with vigorous vortexing. Visible macroscopic agglomerates remained after the vortexing, and the small clusters observed in TEM images most likely represented small fragments of a larger network. Connected micelles have been previously reported for double solvophobic BAB triblock copolymers as a result of the phase separation of B‐blocks in into separate core domains.[^28^, ^35^] Similarly, the sphere clusters and high viscosity in our system were hypothesised to arise from backbone bridges and entanglements resulting in physical crosslinks. These crosslinks may be expected to span across the whole reaction volume, forming a three‐dimensional network of connected spheres.

In reactions targeting long grafts chain entanglements were greatly reduced and macroscopic gelation did not occur. At extremely short graft lengths grafts may have retained sufficient mobility to allow particle clusters to break apart under shear.

Similar dispersion to a gel‐like transitions and TEM findings were observed for all five backbones regardless of their length or grafting density. Partial separation of solvent from the polymer phase was observed at shorter graft lengths when using shorter backbones. For backbones of similar length but different grafting density the gel‐like consistency was reached at similar graft lengths (DP≈10) as opposed to similar *n*_BzMA_/*n*_LMA_ ratios (Figure S14), suggesting the mobility of pBzMA grafts to be a predominant factor in this transition. The formation of precipitates in 10 wt% reactions with **3 b** suggested intermolecular and/or interparticle interactions to play a key role in aggregate stabilisation (Figure S16). Polymerisations targeting a grafting density of 29 % using pLMA_194_‐CTA_29%_ resulted in coagulum even at very short graft lengths (DP 5–12, Figure S18), indicating a practical lower limit for the number of pLMA units per branching point.

### Evaluation of Core Morphologies with SAXS

**Figure 4:**
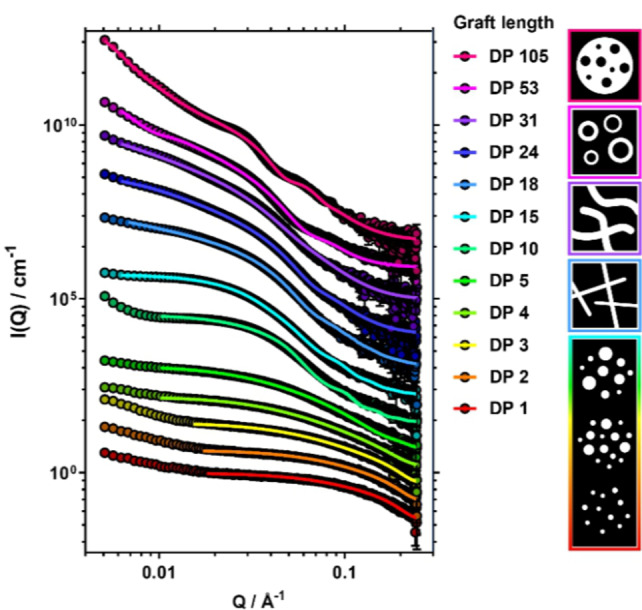
SAXS data (points) and associated structural fits (lines) of PISA reaction mixtures at 20 wt% prepared from pLMA_915_‐CTA_10%_ (1b) to yield pLMA_915_‐*g*‐(pBzMA_*x*_)_*y*_ graft copolymers (6.1–6.12). Graft length increases from bottom to top (data vertically offset for clarity). Scheme illustrates the suggested multicore micelle (DP 1–15), rigid cylinder (DP 18), flexible cylinder (DP 24–31), vesicle (DP 53), and inverse multicore micelle (DP 105) morphologies of the pBzMA cores (in white) against pLMA and n‐dodecane (in black; not to scale).

SAXS was used as a complementary, non‐destructive technique to gain insight into the pBzMA core structure within the achievable *Q* range (0.005–0.24 Å^−1^), thus including the size, composition, and morphology of small features but not the larger structures observed with EM (Figure 4). Data was collected from the reaction mixtures at 25 °C without dilution. Due to the similar chemical compositions and therefore scattering length densities of *n*‐dodecane and the pLMA backbone, solvated pLMA could not be differentiated from the solvent. Therefore, rather than describing the overall particle structure, these data describe the structures formed by the phase separated grafts.

For the shortest grafts (DP 1–5) the data indicated the formation of small spheres with substantially smaller radii (16–18 nm) than the overall particle radii observed by EM (Table S4). The radii were found to increase with increasing DP, with an associated increase in radial polydispersity. An increasing inter‐sphere interaction between the pBzMA cores was indicated by the decreasing value of the “stickiness” parameter of the sticky hard sphere structure factor. This interaction could also be seen in the scattering patterns as a low‐*Q* increase in the scattering intensity, possibly arising from the formation of new cores with increasing solvophobicity and number of grafts. Combining the information obtained from EM and SAXS, the spherical particles observed with EM seemed to have a multicore micellar structure in which grafts of multiple backbones had phase separated into small spherical globules, stabilised by a large pLMA continuum. Backbone entanglements and bridges can be seen to promote the formation of such structures.

A decrease in inter‐core interactions was observed at longer graft lengths (DP 10–15) as suggested by both a larger value of the “stickiness” parameter and a smaller increase in scattering intensity at low‐*Q*. This could be due to increasing solvophobicity of the cores resulting in their further collapse, subsequently stretching of the main chain bridges, and thus a larger inter‐domain distance.

An increase in graft length (DP 18) led to a substantial change in the scattering pattern, particularly at low‐*Q* where the scattered intensity decayed at a rate of *Q* ^−1^ indicating the formation of elongated cylindrical structures. The total length of these rigid cylinders could not be determined within the achievable *Q* range and was therefore fixed for the analysis. The findings suggested a sphere‐to‐cylinder transition to have taken place as a result of change in the packing parameter^[11]^ for each graft‐backbone segment. Similar to the multicore micelles, the cylindrical core domains were thought to exist within a larger pLMA continuum. For longer grafts (DP 24–31) a flexible cylinder form factor was required to obtain adequate fits to these data, suggesting the formation of worm‐like core domains, the radii and Kuhn lengths of which increased with increasing graft length (Table S5).

Another large change in the scattering pattern was observed at DP 53 for which the best fit was obtained using a polydisperse vesicular form factor. The vesicle wall thickness was found to be smaller than the diameter of the cylindrical micelles (Table S6), suggesting greater interdigitation of chains within the bilayer.[^50^, ^51^] Separation of solvent from the polymer phase was observed for this reaction, which could possibly be explained by vesicles growing inwards^[51]^ after the transition had taken place.

Graft DP 105 showed a further change in the scattering pattern. The best fits to these data were obtained with a model describing a large sphere consisting of a pBzMA continuum with smaller spheres of pLMA situated within, implying phase inversion to yield an inverted multicore micelle. With pBzMA now forming the continuous phase within the particle and giving a good contrast against the solvent, the overall particle size and shape could be observed (Table S7). While the total particle radius was found to be close to that observed by TEM (57 nm and 62 nm, respectively), the restricted *Q* range resulted in a lack of an observable Guinier region and therefore the accuracy with which the total radius could be defined by SAXS was limited. Given the stability of these particles in solution, a substantial surface fraction of pLMA must remain. This was supported by the fitting parameters which suggested 25 % of the particle surface to consist of pLMA.

### Time‐Resolved SAXS Study of the Formation of Inverted Multicore Micelles

**Figure 5:**
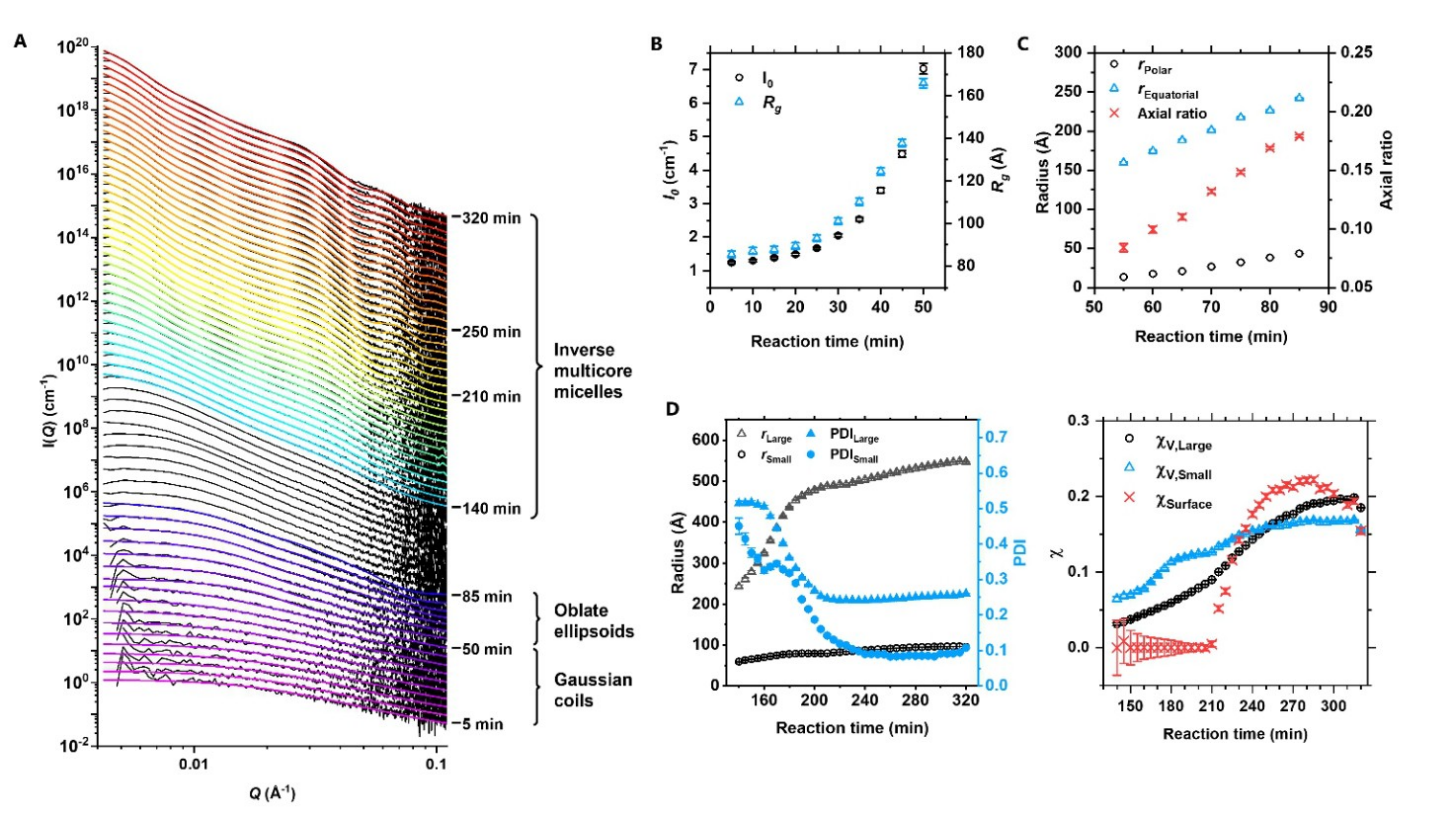
A) Time‐resolved SAXS data (black traces) collected in situ during PISA of pLMA‐*g*‐pBzMA. Fits to the data are shown as coloured lines. Error bars have been omitted for clarity and datasets have been vertically offset. B) Zero‐angle intensity (I_0_) and radius of gyration (*R*_g_) values obtained through fitting data between 5–50 min to Gaussian coil models. C) Equatorial and polar radii (*r*) obtained through fitting data between 55–85 min to ellipsoidal models. D) Radii, radial polydispersities (PDI) and volume fractions (*χ*) obtained through fitting data between 140–320 min to raspberry models describing inverse multicore micelles.

To learn about the transitions leading up to the inverted multicore micellar structure, the polymerisation targeting DP 105 graft was repeated in a time‐resolved SAXS experiment to monitor morphological transitions in situ. Data was collected continuously over 320 min at 70 °C and binned to a 5‐minute time resolution (Figure 5A). An increase in scattering intensity was observed throughout the reaction, indicating successful PISA.

The scattering patterns collected over the first 50 min showed no self‐assembly and suggested that individual molecules remained as Gaussian chains. The increasing radius of gyration (*R*_g_) and an increase in zero‐angle intensity (*I*_0_) were consistent with the growth of the grafts (Figure 5B). After 50 min a change in the gradient of the scattering patterns could be identified at *Q=*0.02 Å^−1^, indicating the onset of a coil‐to‐globule transition. Best fits to these data were obtained using a model describing oblate ellipsoids. A longer reaction time led to a gradual growth of these ellipsoids with the equatorial radius increasing faster than the axial radius, indicating swelling towards a less oblate morphology (Figure 5C). The ellipsoidal phase was relatively short‐lived, suggesting a narrow region within the phase diagram where these structures may be found. Notably, this morphology was not found in the earlier measurements at full monomer conversion and the core volumes of the ellipsoids were substantially larger than those of the spheres in multicore micelles (1 400–13 000 nm^3^ and 17–1 300 nm^3^, respectively). These differences were ascribed to differences in backbone concentrations and therefore chain entanglements, the presence of BzMA in the solvent phase affecting the solubility of grafts at low conversions, and/or measurement temperature.

Between 90–135 min the data lacked distinct features in the scattering patterns, suggesting a high polydispersity of the particles or the presence of multiple interacting form factors. Similar observations have been made in previous PISA studies.[^51^, ^52^] After 140 min two minima were identified at *Q*=0.02 and 0.05 Å^−1^, consistent with the previously encountered inverse multicore micelle morphology. Fitting these data indicated the radii of the large pBzMA continuum and the small pLMA pockets to grow with an increasing reaction time, as well as an increase in the volume fraction and a decrease in the radial polydispersity of both components (Figure 5D). While the increasing radius and volume fraction of the pBzMA phase could be explained by an increasing graft length, the reason behind the increase in volume fraction of the pLMA phase was less evident. A possible explanation could be a change in the solvation of the solvophilic pLMA domains—now embedded within the particle interior—resulting in their swelling or fusion. The surface fraction of pLMA remained close to zero throughout this first phase. This observation could provide an explanation for why the previously discussed DP 50 reaction had a “dry” consistency, indicative of solvent encapsulation within the particles. While the transition from ellipsoids to the inverted structure remains unclear based on this experiment alone, the final morphology could be hypothesised to have evolved through an ellipsoid fusion‐type of pathway.^[53]^

## Conclusion

The PISA of pLMA‐*g*‐pBzMA graft copolymers in *n*‐dodecane was shown to result in the formation of spherical particles or clusters thereof, as opposed to the spherical and worm‐like aggregates obtained with their linear diblock copolymer analogue. The physical constraints of the branched architecture promoted the formation of multicore morphologies—uncommonly encountered in PISA studies. Two critical structural parameters of the polymers could be identified in this study: the backbone concentration which influenced the degree of entanglements and thus macroscopic gelation, and the targeted graft length which determined the core morphology.

It may be anticipated that the PISA of graft copolymers conducted through *grafting from* polymerisations, regardless of the copolymer/solvent system, will generally be strongly influenced by chain entanglements if the grafting density is sufficiently low for the backbone to retain some flexibility and if the backbone is long and used in high concentration. Our observations are expected to differ significantly from the PISA behaviour of rigid, densely grafted polymers with reduced chain entanglements—an area which remains unexplored and an intriguing subject for future studies.

## Conflict of interest

The authors declare no conflict of interest.

1

## Supporting information

As a service to our authors and readers, this journal provides supporting information supplied by the authors. Such materials are peer reviewed and may be re‐organized for online delivery, but are not copy‐edited or typeset. Technical support issues arising from supporting information (other than missing files) should be addressed to the authors.

Supporting Information

## Data Availability

The data that support the findings of this study are available from the corresponding author upon reasonable request.
