# Supplementary material for: Polymerisation‐Induced Self‐Assembly of Graft Copolymers
Source: Angew Chem Int Ed Engl. 2022 Sep 29;61(44):e202210518. doi: 10.1002/anie.202210518 (PMC9828155; doi:10.1002/anie.202210518)
Supplement: Supplementary file 1 — Supporting Information [file ANIE-61-0-s001.pdf]

Angewandte Chemie
Eine Zeitschrift der Gesellschaft Deutscher Chemiker

Supporting Information

Polymerisation-Induced Self-Assembly of Graft Copolymers

*S. Häkkinen, J. Tanaka, R. Garcia Maset, S. C. L. Hall, S. Huband, J. Y. Rho, Q. Song, S. Perrier**

Table of Contents

1 Instrumental Methods ... 2
1.1 Nuclear Magnetic Resonance Spectroscopy ... 2
1.2 Size Exclusion Chromatography ... 2
1.3 Small-Angle X-Ray Scattering ... 2
1.3.1 Graft length series ... 3
1.3.2 Time-resolved SAXS experiment ... 3
1.4 Scanning Electron Microscopy ... 3
1.5 Transmission Electron Microscopy ... 3
2 Materials ... 4
3 Synthetic Methods and Characterisation Data ... 5
3.1 Preparation of 4-Cyano-4-(((dodecylthio)carbonothioyl)thio)pentanoic Acid ... 5
3.2 Preparation of Poly(lauryl methacrylate-*stat*-hydroxyethyl methacrylate) Copolymers ... 6
3.3 Functionalisation of Poly(lauryl methacrylate-*stat*-hydroxyethyl methacrylate) Copolymers ... 10
3.4 "Grafting From" Dispersion Polymerisation of Benzyl Methacrylate ... 13
3.4.1 Effect of Graft Length ... 15
3.4.2 Effect of Main Chain Length ... 19
3.4.3 Effect of Grafting Density ... 21
3.4.4 Effect of Concentration ... 23
4 References ... 27

1 Instrumental Methods

1.1 Nuclear Magnetic Resonance Spectroscopy

^{1}H Nuclear Magnetic Resonance (NMR) spectra and ^{1}H-^{13}C Heteronuclear Single Quantum Coherence (HSQC) spectra were recorded in deuterated chloroform ($CDCl_3$) on Bruker Avance III HD (300 MHz or 400 MHz) spectrometer at 300 K. Chemical shift values (δ) are reported in ppm. Tetramethylsilane (TMS) was used as the internal standard.

1.2 Size Exclusion Chromatography

Size Exclusion Chromatography (SEC) was carried out using an Agilent Infinity II MDS instrument equipped with differential refractive index (DRI), viscometry (VS), dual angle light scattering (DALS) and multiple wavelength UV detectors. The system was equipped with 2×PLgel Mixed C columns (300×7.5 mm, 200 to 200,000 g/mol operating range) and a PLgel 5 µm guard column. Analyte samples were prepared in $CHCl_3$ and filtered through a Fisherbrand PTFE syringe filter with 0.2 µm pore size before injection. Samples were run in $CHCl_3$ at 1 ml/min at 30 °C. Experimental number-average molar mass ($M_{n,SEC}$) and dispersity ($Đ$) values of synthesised polymers were determined with Agilent GPC/SEC software by using Agilent EasyVial poly(methyl methacrylate) (PMMA) calibration.

1.3 Small-Angle X-Ray Scattering

Small-Angle X-ray Scattering (SAXS) measurements were performed using a Xenocs Xeuss 2.0 equipped with a micro-focus Cu K_α source collimated with scatterless slits providing a 0.8 mm diameter beam. SAXS patterns were recorded using a Pilatus 300K detector with a pixel size of 0.172 mm×0.172 mm. The sample to detector distance was calibrated using silver behenate ($AgC_{22}H_{43}O_2$) providing a value of 2.481(5) m, providing an effective scattering vector Q range of 0.005-0.24 Å^{-1}, where Q is defined as

$$Q = \frac{4\pi \sin\theta}{\lambda}\ , \tag{3.2}$$

where 2θ is the scattering angle and λ is the X-ray wavelength. Reaction mixtures were mounted at 20 wt% without dilution in a 1 mm (Ø) borosilicate glass capillaries or a Perspex holder with Kapton tape if too viscous for the capillaries. Data were collected for 20 min at 25 °C unless otherwise stated. A radial integration of the 2D scattering profile was performed using FOXTROT software and the resulting data corrected for the absorption, sample thickness and background.[1] Finally, the scattering intensity was then rescaled to absolute intensity using glassy carbon as a standard.[2]

SAXS data were analysed using model-dependent analysis implemented in SasView software.[3] The scattering length density (SLD) defining the "scattering power" of a material, is defined as the sum of X-ray scattering lengths, b_i, of N atoms within a given molecular or particle volume, V_m, as given by

$$\mathrm{SLD} = \frac{\sum_{i=1}^{N} b_i}{V_m}\ . \tag{3.3}$$

The SLD of a material can also be calculated using the bulk density ρ, atomic molar mass M_i and Avogadro's constant N_A,[4] where

$$\mathrm{SLD} = \frac{\rho N_A \sum_{i=1}^{N} b_i}{\sum_{i=1}^{N} M_i}. \tag{3.4}$$

In this study, the SLD of dodecane, LMA and BzMA were calculated as 7.41×10^{-6} Å^{-2}, 8.22×10^{-6} Å^{-2}, and 9.52×10^{-6} Å^{-2}, respectively, and fixed for the fitting procedure.

1.3.1 *Graft length series*

Graft DP 1 to 15 were analysed using a spherical form factor with a Gaussian radial polydispersity applied and a sticky hard sphere structure factor (**Table S4**).[5, 6] Graft DP 18 was analysed using a cylindrical form factor with a Gaussian radial polydispersity applied.[7] Graft DPs 24 and 37 were analysed using a flexible cylinder form factor, describing a cylinder with a total persistence length which can be split into shorter segments which can be considered rigid, described by the Kuhn length (**Table S5**).[8, 9] Similarly to the cylindrical form factor described above, a Gaussian radial polydispersity was also applied to the flexible cylinder model. Graft DP 53 was analysed using a vesicular form factor with parameters describing the wall thickness and vesicle radius (**Table S6**)[6] Similarly to the spherical form factors, a Gaussian polydispersity was also applied to the wall thickness and vesicle radius. Finally, graft DP 100 was analysed using a "raspberry" form factor,[10] describing small spheres within a larger spherical structure (**Table S7**). In this case, the SLD of the small spheres was fixed to that for LMA, and the SLD of the larger spheres fixed to that for BzMA. The fractional penetration depth of small spheres within the larger spheres was set to 1, representing small spheres distributed throughout the larger sphere. Similar to models above, a Gaussian radial polydispersity was applied.

1.3.2 *Time-resolved SAXS experiment*

The reaction carried out in the time-resolved SAXS experiment was equivalent to (**6.12**) conducted at 20 wt% solids targeting a graft length of 106 repeating units. Reaction mixture was prepared in a septum-capped vial by dissolving $pLMA_{915}$-$CTA_{10\%}$ (**6**) (10.72 mg, 3.6 µmol side chain CTAs) and BzMA (70.88 mg, 386 µmol) in n-dodecane (412 µl). V-601 initiator stock solution (23 µl, 0.10 µmol, 1.0 mg/ml in n-dodecane) was added. The reaction mixture was deoxygenated by bubbling N_2 into the solution for 15 min and transferred into a 1 mm (Ø) borosilicate glass capillary under an argon blanket. The capillary was kept at room temperature, in the dark and under argon until mounting (<2 h). The capillary was mounted at room temperature, aligned, and then heated up to 70 °C at the rate of 5 °C/min to start the reaction using a Linkam HFSX 350 temperature stage. Data were collected at 70 °C continuously over 320 min and binned to a 5 min time resolution.

Data collected from 5 to 50 min were fitted using a Gaussian coil form factor describing individual polymer chains in terms of the zero-angle intensity, I_0, proportional to the volume fraction of polymer chains in solution, the volume of an individual chain and the SLD contrast between polymer and solvent, and the radius of gyration, R_g.[11] Data from 55 to 85 min were fitted using an ellipsoidal form factor with parameters describing the radii along the polar and equatorial axes.[12] Data collected from 140 to 320 min were fitted using the raspberry form factor as described above.

1.4 Scanning Electron Microscopy

Scanning electron microscopy (SEM) was carried out using a Zeiss SUPRA 55-VP instrument operating at 2-12 kV accelerating voltage. Polymer samples were spin-coated or merely deposited onto silicon wafers directly from reaction mixtures and placed under vacuum overnight. Samples were coated with carbon using an Emitech K950X turbo-pumped evaporator prior to imaging.

Cryo-SEM images were taken on a Zeiss Supra 55VP fitted with a Gatan Alto 2500 cryo transfer system.

1.5 Transmission Electron Microscopy

Transmission electron microscopy (TEM) was conducted using a JEOL 2100Plus instrument operating at 80-200 kV and equipped with a Gatan Orius 11-megapixel digital camera. Samples were deposited directly from reaction mixtures unless otherwise stated onto 300 mesh carbon-coated copper grids. Excess sample was blotted off with a filter paper.

2 Materials

Chloroform-*d* ($CDCl_3$, 99.8 atom % D) and dichloromethane anhydrous (DCM, ≥99.8%) were purchased from Sigma-Aldrich. 1,1'-Azobis(cyclohexane-carbonitrile) (V-40, 98%), carbon disulfide (CS_2, ≥99.9%), and 1-dodecanethiol (≥98%) were purchased from Aldrich. *N,N*-Dicyclohexylcarbodiimide (DCC, 99%) and iodine were purchased from Acros Organics. 4,4'-Azobis(4-cyanovaleric acid) (ACVA, 98%), 4-(dimethylamino)pyridine (DMAP, ≥99.0%), and dodecane (99+%) were purchased from Alfa Aesar. Diethyl ether, ethyl acetate, and toluene were purchased from Merck. Sodium hydroxide was purchased from Fisher Scientific. Dimethyl 2,2'-azobis(2-methylpropionate) (V-601) was purchased from Wako Chemicals.

Benzyl methacrylate (BzMA, 96%), 2-hydroxyethyl methacrylate (HEMA, >99%), and lauryl methacrylate (LMA, 96%) were purchased from Sigma-Aldrich and passed through neutral aluminium oxide to remove inhibitors prior to use.

Methyl 4-cyano-4-(((dodecylthio)carbonothioyl)thio)pentanoate (MCPDTC) had previously been synthesised by the Perrier group using a reported protocol was and verified spectroscopically prior to use.[13] ^{1}H NMR (400 MHz, $CDCl_3$, δ): 0.88 (t, 3H, -C$\underline{H}_3$), 1.19-1.35 (br s + m, 16H, -CH_2-(C$\underline{H}_2$)$_8$-CH_3), 1.40 (m, 2H, -S-CH_2-CH_2-C$\underline{H}_2$-), 1.70 (m, 2H, -S-CH_2-C$\underline{H}_2$-), 1.89 (s, 3H, -S(CN)C$\underline{H}_3$-), 2.32-2.62 (m, 2H, -OOC-C$\underline{H}_2$-CH_2-), 2.63 (t, 2H, -OOC-CH_2-C$\underline{H}_2$-), 3.33 (t, 2H, -S-C$\underline{H}_2$-), 3.71 (s, 3H, $\underline{H}_3$C-COO-).

3 Synthetic Methods and Characterisation Data

3.1 Preparation of 4-Cyano-4-(((dodecylthio)carbonothioyl)thio)pentanoic Acid

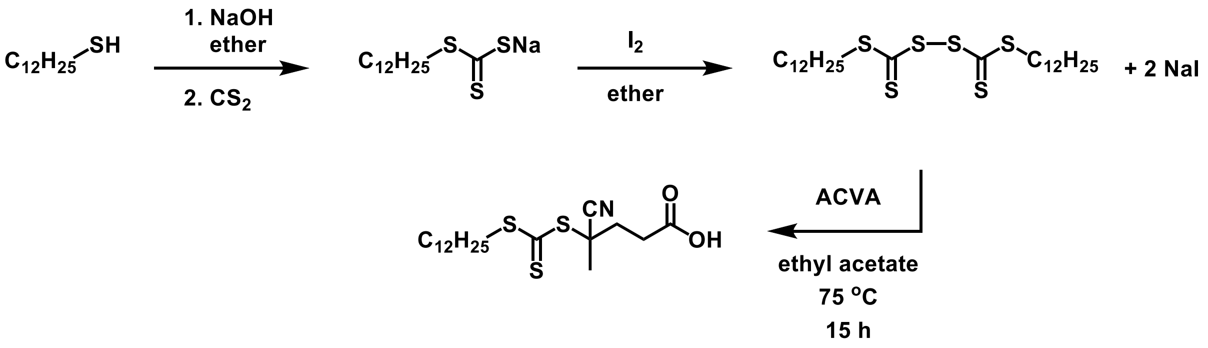

Scheme S1 Synthetic route used for the preparation of CPADTC.

4-Cyano-4-(((dodecylthio)carbonothioyl)thio)pentanoic acid (CPADTC) RAFT agent was prepared by adapting a previously reported protocol (**Scheme S1**).[14] Ground NaOH (6.23 g, 0.16 mol) was suspended in 500 ml diethyl ether using an overhead stirrer. 1-Dodecanethiol (30.0 g, 0.15 mol) was added dropwise and stirring was continued for 10 min. Carbon disulfide (11.9 g, 0.16 mol) was added in one shot and stirring was continued for 1 h. Reaction mixture was cooled down in the freezer and solids were filtered, washed with cold diethyl ether and dried, giving sodium dodecyl carbonotrithioate as a yellow solid (25.2 g, 57%). The product was used in the next step without purification.

Sodium dodecyl carbonotrithioate (12.5 g, 42 mmol) was suspended in 250 ml diethyl ether using an overhead stirrer. Solid iodine (5.38 g, 21 mmol) was added portionwise and stirring was continued for 1 h. Salts were filtered off and the brown filtrate was washed with aqueous sodium thiosulfate (0.4 M, 3x), deionized water (1x) and brine (1x). The organic layer was dried over $MgSO_4$ overnight and solvent was removed to give bis-(dodecylsulfanylthiocarbonyl) disulfide an orange solid (9.20 g, 17 mmol, 80%).

Bis-(dodecylsulfanylthiocarbonyl) disulfide (9.20 g, 17 mmol) was dissolved in 250 ml ethyl acetate and 4,4′-azobis(4-cyanopentanoic acid) (6.97 g, 25 mmol) was added. The mixture was refluxed at 75 °C for 15 h. Solids were filtered off, ethyl acetate was removed and 200 ml dichloromethane was added. The organic layer was washed with deionized water (5x), brine (1x), and the product was dried over $MgSO_4$. Volatiles were removed, product was recrystallized from hexane and dried under vacuum yielding CPADTC as a pale yellow solid (6.30 g, 16 mmol, 47%). ^1H NMR (400 MHz, $CDCl_3$, δ): 0.88 (t, 3H, -C***H***$_3$), 1.19-1.35 (br s + m, 16H, $-CH_2-(C\boldsymbol{H_2})_8-CH_3$), 1.40 (m, 2H, $-S-CH_2-CH_2-C\boldsymbol{H_2}-$), 1.70 (m, 2H, $-S-CH_2-C\boldsymbol{H_2}-$), 1.89 (s, 3H, $-S(CN)C\boldsymbol{H_3}-$), 2.32-2.62 (ddd, 2H, $HOOC-C\boldsymbol{H_2}-$), 2.69 (t, 2H, $HOOC-CH_2-C\boldsymbol{H_2}-$), 3.33 (t, 2H, $-S-C\boldsymbol{H_2}-$).

3.2 Preparation of Poly(lauryl methacrylate-*stat*-hydroxyethyl methacrylate) Copolymers

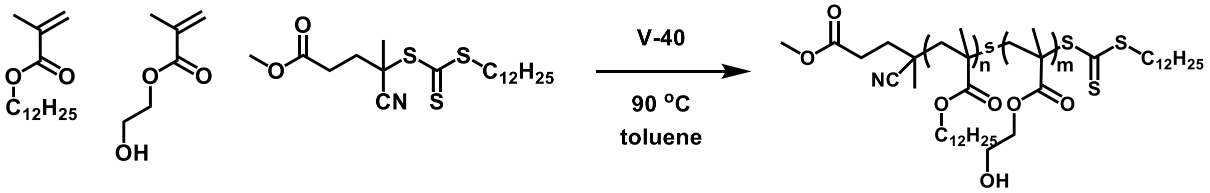

Scheme S2 RAFT solution polymerisation of LMA and HEMA used in this work to construct graft copolymer main chains.

A series of poly(lauryl methacrylate-*stat*-hydroxyethyl methacrylate) (p(LMA-*s*-HEMA)) copolymers (**1-5**) were prepared using RAFT polymerisation (**Scheme S2**) with the following general procedure. For preparing main chain (**1**), MCPDTC (11.90 mg, 0.289 mmol), HEMA (394 mg, 3.03 mmol), LMA (7.00 g, 27.5 mmol) and toluene (6.18 ml, $[M]_0$ = 2 M) were added in a 25 ml glass vial. V-40 initiator was added as a stock solution (20 mg/ml in toluene, $[CTA]_0/[I]_0$ = 5). The vial was sealed with a rubber septum and N_2 gas was bubbled into solution for 20 min. Reaction was initiated by immersing the vial in a pre-heated oil bath set to 90 °C. (**Figure S3**). Reaction was stopped at 70-98% conversion after ≥14 h by cooling down to room temperature. The copolymers were not isolated prior to the functionalisation step. A portion of polymer (**1**) was precipitated in MeOH thrice to obtain a ^{1}H NMR spectrum of an isolated product (**Figure S4**).

Monomer conversion was determined using ^{1}H NMR spectroscopy by setting the area δ = 3.5-4.5 ppm as a constant and quantifying the disappearance of the vinyl signals, δ_{LMA} = 5.5 ppm and δ_{HEMA} = 5.6 ppm. SEC samples were sampled directly from reaction mixtures.

Theoretical number-average molar masses ($M_{\mathrm{n,th}}$) were calculated as

$$M_{\mathrm{n,th}} = M_{\mathrm{CTA}} + \frac{[\mathrm{LMA}]_0}{[\mathrm{CTA}]_0} p_{\mathrm{LMA}} M_{\mathrm{LMA}} + \frac{[\mathrm{HEMA}]_0}{[\mathrm{CTA}]_0} p_{\mathrm{HEMA}} M_{\mathrm{HEMA}} \qquad \text{(S1)}$$

where $[\mathrm{LMA}]_0$, $[\mathrm{HEMA}]_0$, and $[\mathrm{CTA}]_0$, are the initial LMA, HEMA, and CTA concentrations, respectively, p is the monomer conversion as determined by ^{1}H NMR spectroscopy and M_{LMA}, M_{HEMA}, and M_{CTA} are the molar masses of LMA, HEMA, and the CTA, respectively.

All main chains and their characteristics are listed in **Table S1**.

Table S1 Main chain copolymers employed in this study.

	Structure [A]	Conv. (%)		*t*	DP$_{\mathrm{tot}}$ [B]	n_{HEMA}/DP$_{\mathrm{tot}}$ [C]	$M_{\mathrm{n,th}}$ [D]	$M_{\mathrm{n,SEC}}$ [E]	*Đ* [E]
		LMA	HEMA	(h)		(%)	(g/mol)	(g/mol)	
**(1)**	p(LMA$_{816}$-*s*-HEMA$_{99}$)	88	96	26	915	11	221,000	152,000	1.38
**(2)**	p(LMA$_{424}$-*s*-HEMA$_{50}$)	90	97	22	474	11	115,000	80,400	1.24
**(3)**	p(LMA$_{182}$-*s*-HEMA$_{24}$)	76	94	22	206	12	49,800	40,700	1.16
**(4)**	p(LMA$_{844}$-*s*-HEMA$_{52}$)	84	99	14	896	5.9	222,000	140,000	1.32
**(5)**	p(LMA$_{914}$-*s*-HEMA$_{25}$)	88	98	15	939	2.7	236,000	143,000	1.37

[A] Number of repeating units were calculated based on conversion as given by ^{1}H NMR ($CDCl_3$). [B] Theoretical average number of monomer units per chain, $n_{\mathrm{LMA}} + n_{\mathrm{HEMA}}$. [C] Theroretical HEMA content. [D] Theoretical number-average molar mass as given by conversion (**Eq S1**). [E] Experimental number-average molar mass and dispersity as given by SEC analysis in $CHCl_3$ with DRI detection and PMMA calibration.

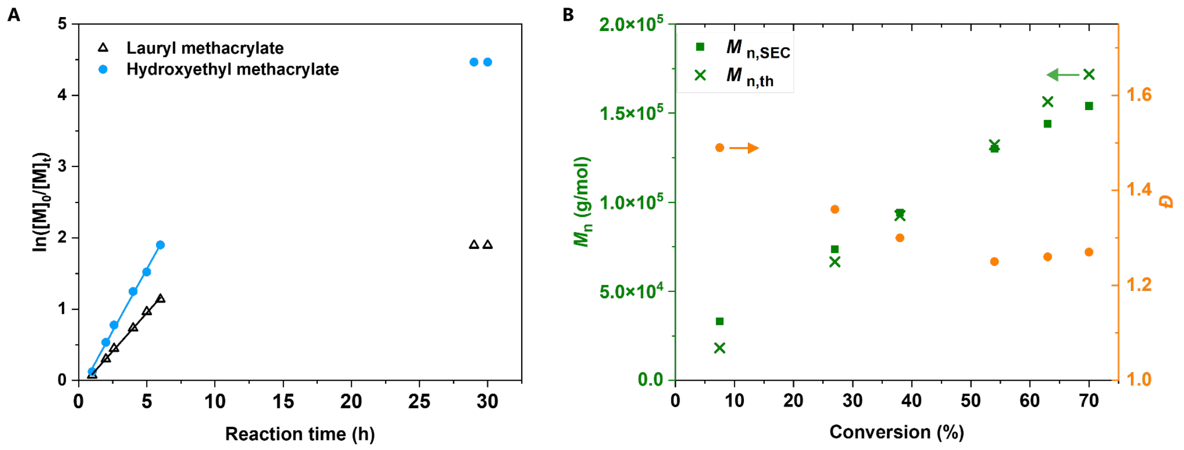

Figure S1 Copolymerisation kinetics and molecular weight evolution for a reaction conducted under identical conditions to those used in the synthesis of p(LMA_{816}-*s*-$HEMA_{99}$) A) Reaction kinetics and a linear fit (r^2_{LMA} > 0.99, r^2_{HEMA} > 0.99). B) Evolution of experimental ($M_{n,SEC}$) and theoretical ($M_{n,th}$) molecular weights and dispersity with increasing monomer conversion. $M_{n,SEC}$ was obtained by SEC in $CHCl_3$ using DRI detection and PMMA calibration.

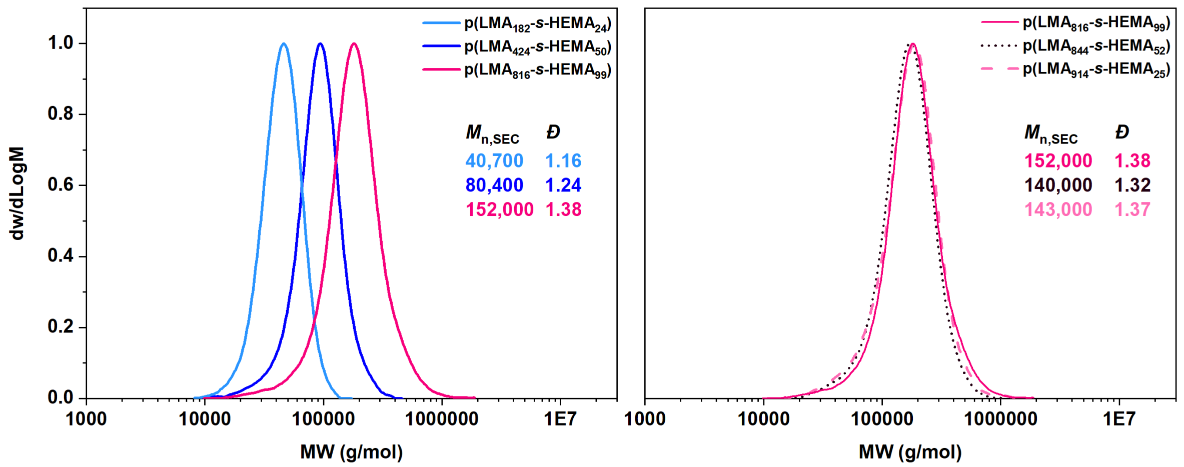

Figure S2 Size exclusion chromatograms of the main chain copolymers in $CHCl_3$ as given by DRI detection and PMMA calibration. Left: Three main chains of dissimilar lengths (DP 206-915) and similar HEMA contents (11-12%) (**1**-**3**). Right: Three main chains with dissimilar HEMA contents (3-12%) but similar lengths (DP 896-939) (**1**, **4**, and **5**).

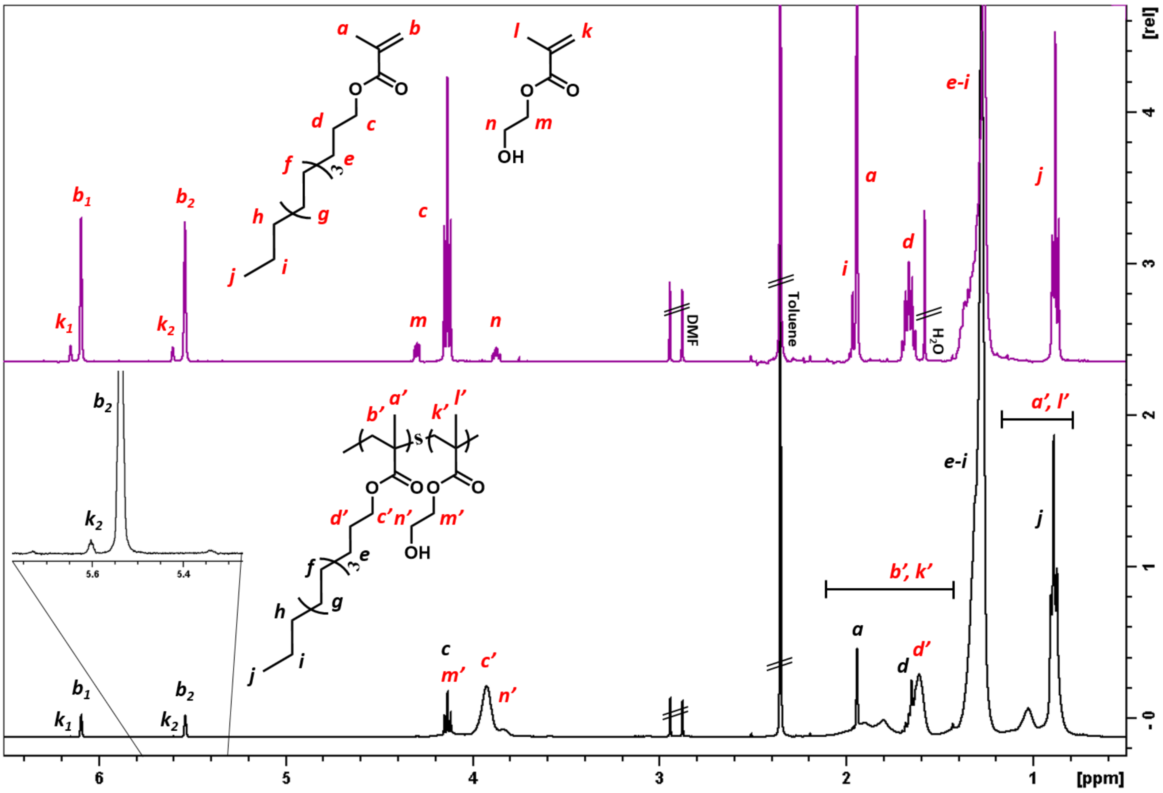

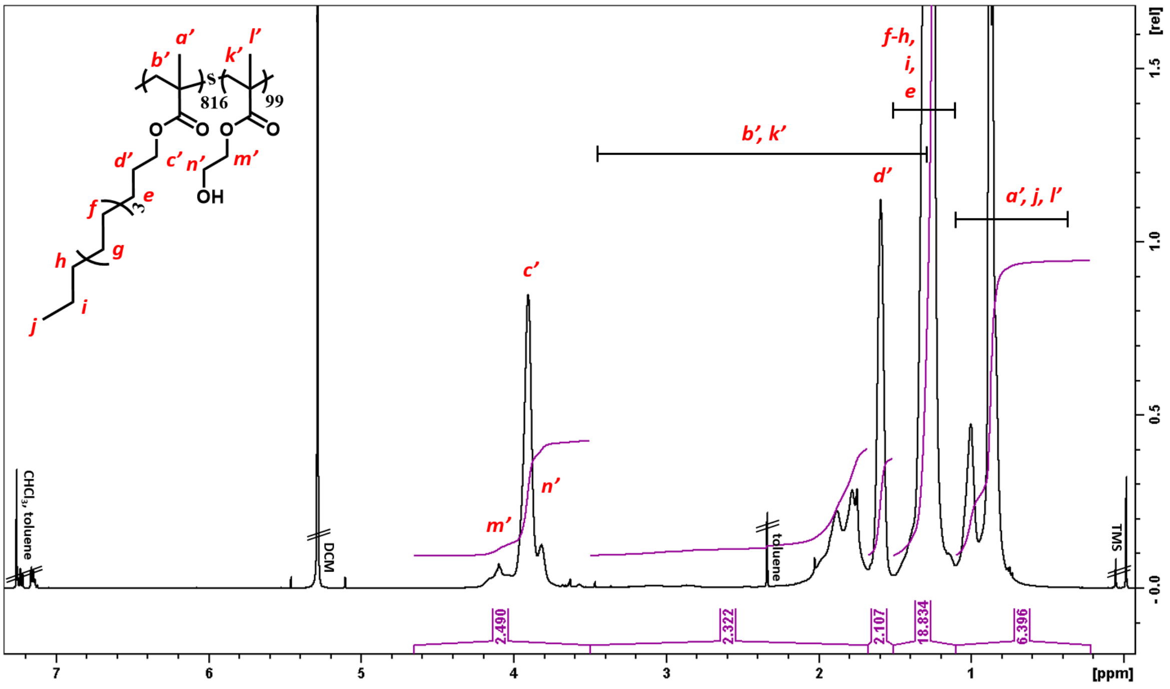

Figure S3 Representative ^{1}H NMR spectra (400 MHz, $CDCl_3$) for main chain copolymerisations. Top: Spectra obtained before and after reaction for conversion monitoring. Bottom: Isolated copolymer p(LMA_{816}-*s*-$HEMA_{99}$) (**1**).

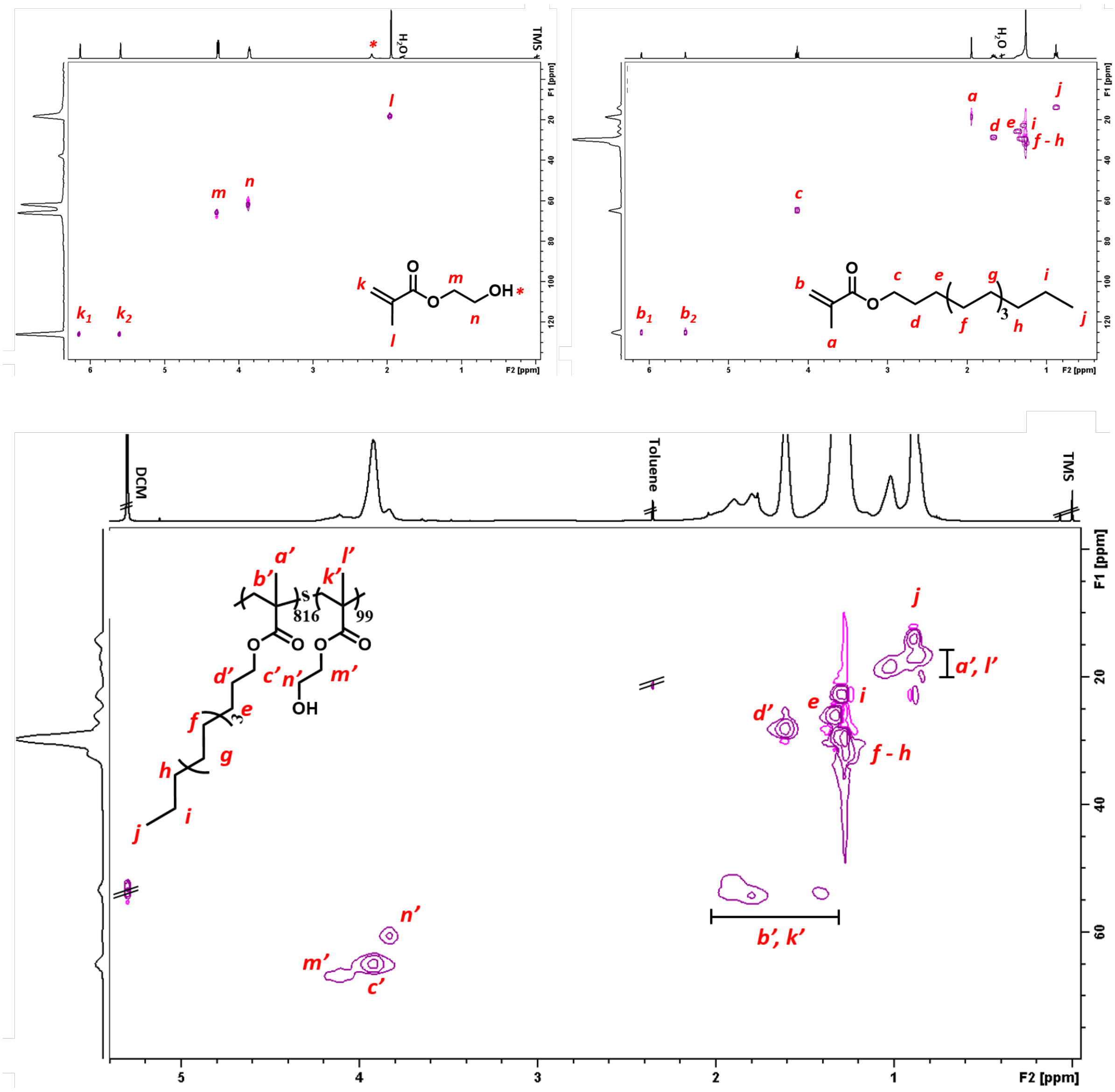

Figure S4 Representative ^{1}H-^{13}C HSQC spectra (400 MHz, $CDCl_3$) for main chain copolymer synthesis. Top: Spectra for the main chain monomers, HEMA and LMA. Bottom: Isolated copolymer p(LMA_{816}-*s*-$HEMA_{99}$) (**1**).

3.3 Functionalisation of Poly(lauryl methacrylate-*stat*-hydroxyethyl methacrylate) Copolymers

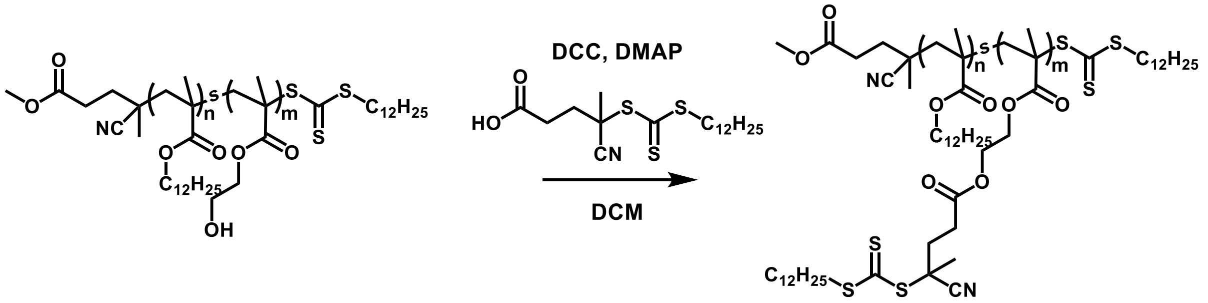

Scheme S3 Synthetic route used in this work for main chain functionalisation.

The Steglich esterification (**Scheme S3**) was used with the following general procedure to couple CPADTC onto copolymer main chains (**1**-**5**) to yield functionalised main chains (**6**-**10**) (**Table S2**). An aliquot of the polymer solution from the copolymerisation step (4.00 g p(LMA_{424}-*s*-$HEMA_{50}$) (**2**), 2.1 mmol HEMA) was added to a dry 250 ml round-bottom flask and toluene was removed by rotary evaporation at room temperature. CPADTC (1.26 g, 3.1 mmol) and a stir bar were added, and the flask was sealed with a septum. Anhydrous DCM (125 ml) was cannulated into the flask and the mixture was stirred at room temperature until the polymer and CPADTC had fully dissolved. DMAP (25 mg, 0.2 mmol) was added as a solid and the flask was immersed in an ice bath. Solid DCC (0.612 g, 3.0 mmol) was added to start the reaction and stirring was continued for 15 minutes over ice, during which dicyclohexylurea started to precipitate out of solution. The flask was taken off ice and stirring was continued overnight. The reaction mixture was filtered twice through cotton wool and concentrated using a rotary evaporator at room temperature. The product was precipitated into methanol, centrifuged, isolated, and redissolved in DCM. Precipitations ($n \geq 3$) were carried out until no free CTA remained in the product.

The polymers were characterised with ^{1}H NMR spectroscopy (**Figure S6**) and SEC (**Figure S5**).

The extent of functionalisation, or the maximum number of grafting points per main chain (n_{CTA}), was calculated from the ^{1}H NMR spectra of the isolated polymers as

$$n_{\text{CTA}} = \frac{n_{\text{CPADTC,NMR}}}{n_{\text{CPADTC,ideal}}} n_{\text{HEMA}} = \frac{\dfrac{\int \mathrm{H}_H}{\int \mathrm{H}_{c'+n''+m''}}}{\dfrac{2n_{\text{HEMA}}}{4n_{\text{HEMA}} + 2n_{\text{LMA}}}} n_{\text{HEMA}}, \quad \text{(S2)}$$

where n_{HEMA} and n_{LMA} are the number of HEMA and LMA units, respectively, as given by conversion. The degree of functionalisation ($n_{\text{CTA\%}}$) of the main chain was defined as

$$n_{\text{CTA\%}} = \frac{n_{\text{CTA}}}{\text{DP}_{\text{tot}}} \cdot 100 = \frac{n_{\text{CTA}}}{\text{DP}_{\text{LMA+HEMA}}} \cdot 100. \quad \text{(S3)}$$

Theoretical number-average molar masses ($M_{\text{n,th}}$) of the functionalised main chains, $\text{pLMA}_{\text{DP,tot}}$-CTA$n_{\text{CTA\%}}$, were calculated as

$$M_{\text{n,th}} = M_{\text{pre,th}} + n_{\text{CTA}} M_{\text{CPADTC}} \quad \text{(S4)}$$

where $M_{\text{pre,th}}$ is the theoretical molar mass of the precursor copolymer, p(LMA-*s*-HEMA) (**Table S1**), and M_{CPADTC} is the molar mass of CPADTC.

Table S2 Functionalised main chain copolymers employed in this study.

	Structure [A]	Functionalised [B] HEMA (%)	n_{CTA} [C]	$M_{n,th}$ [D] (g/mol)	$M_{n,SEC}$ [E] (g/mol)	$Đ$ [E]
**(6)**	$pLMA_{915}$-$CTA_{9\%}$	82	82	254,000	207,000	1.48
**(7)**	$pLMA_{474}$-$CTA_{10\%}$	89	46	133,000	114,000	1.28
**(8)**	$pLMA_{206}$-$CTA_{9\%}$	75	19	57,200	55,900	1.16
**(9)**	$pLMA_{896}$-$CTA_{4\%}$	66	36	236,000	175,000	1.41
**(10)**	$pLMA_{939}$-$CTA_{2\%}$	63	17	243,000	162,000	1.36

[A] The *x* and *y* in the formula $pLMA_x$-CTA_y indicate the DP of the main chain (DP_{tot}) and the degree of functionalisation ($n_{CTA\%}$, **Eq S3**), respectively. [B] Degree of functionalisation of HEMA repeating units based on the ^{1}H NMR spectrum of the product and the theoretical structure of the precursor copolymer. [C] Number of CPADTC units per polymer (**Eq S2**) [D] Calculated based on the structure of the precursor copolymer (**1-5**) and number of CPADTC units (**Eq S4**). [E] Experimental number-average molar mass and dispersity as given by SEC analysis in $CHCl_3$ with DRI detection and PMMA calibration.

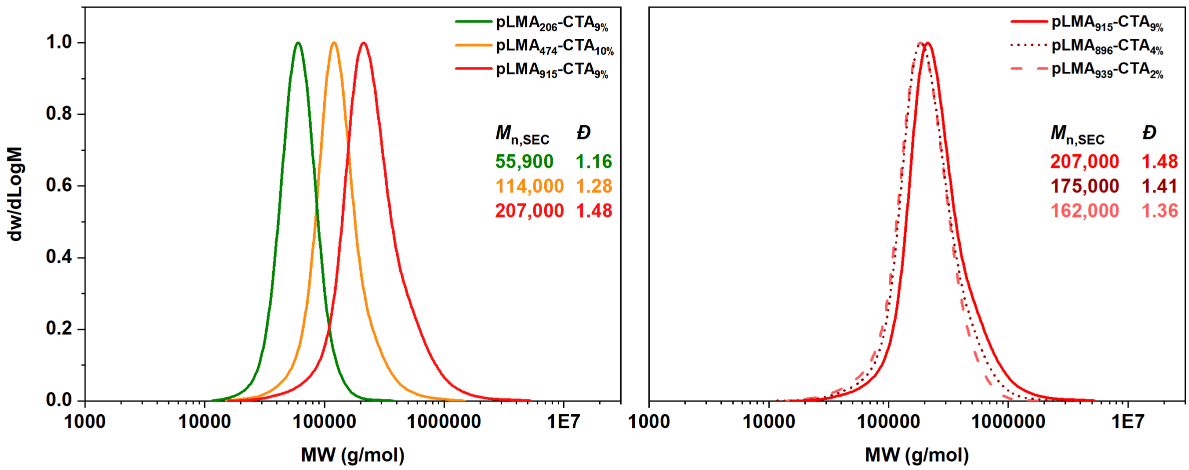

Figure S5 Size exclusion chromatograms of the functionalised main chain copolymers in $CHCl_3$ as given by DRI detection and PMMA calibration. Left: Three main chains of dissimilar lengths (DP 206-915) and similar degrees of functionalisation (10-11%) (**6-8**). Right: Three main chains of similar lengths (DP 896-939) but dissimilar degrees of functionalisation (3-12%) (**6**, **9**, and **10**).

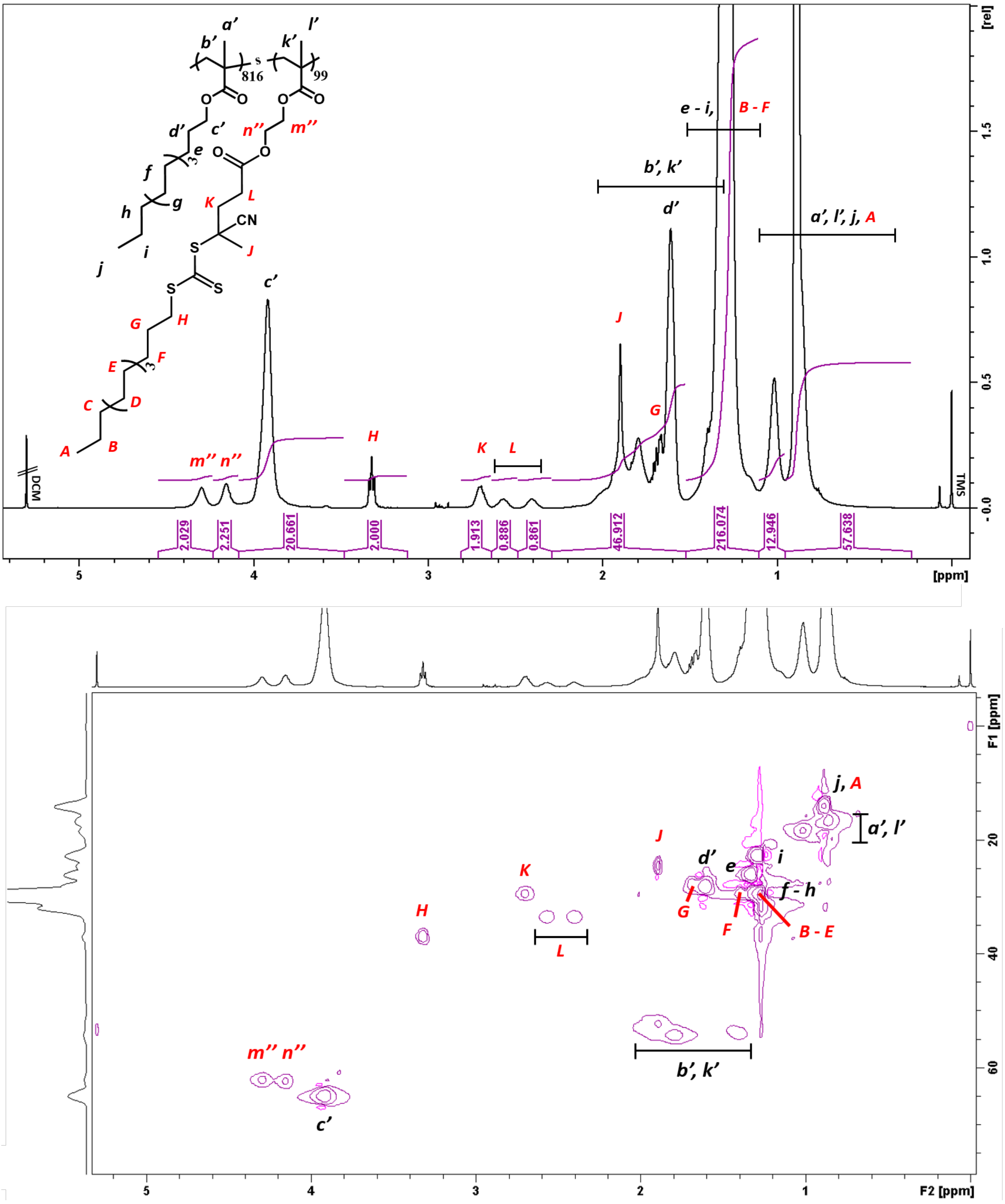

Figure S6 Representative ^{1}H NMR and ^{1}H-^{13}C HSQC spectra (400 MHz, 100 mg/ml in $CDCl_3$) of functionalised main chain $pLMA_{915}$-$CTA_{10\%}$ (**6**).

3.4 "Grafting From" Dispersion Polymerisation of Benzyl Methacrylate

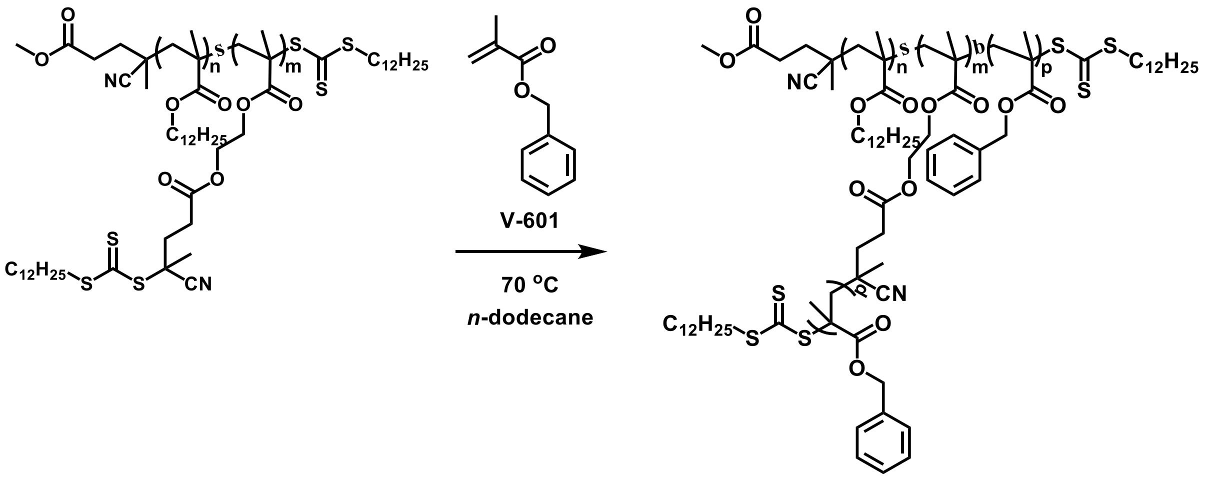

Scheme S4 Dispersion polymerisation conditions used in this work for studying PISA of graft copolymers.

All dispersion polymerisations of BzMA were carried out at 10 wt% or 20 wt% concentration. The following general procedure and $[CTA]_0/[I]_0$ = 40 was used for all reactions. For targeting a graft length of 5 repeating units at 20 wt% using $pLMA_{915}$-$CTA_{10\%}$ (**6**), a stock solution of $pLMA_{915}$-$CTA_{10\%}$ (211 mg, 32.2 wt% in dodecane, 23.0 µmol side chain CTA) was weighed into a 2 ml septum screw-cap vial. BzMA (23.50 mg, 128 µmol), dodecane (145 µl), and V-601 stock solution (1.0 mg/ml in dodecane) were added, the mixture was homogenised thoroughly using a roller mixer, and purged with nitrogen through the septum for 10 min. The vial was immersed in a pre-heated oil bath set at 70 °C. Reactions were carried out for 12 h without stirring and stopped by letting the solutions cool down to room temperature. Resulting materials were stored at room temperature (20-30 °C).

The graft copolymers were characterised using ^{1}H NMR spectroscopy and SEC by sampling directly from the reaction mixture.

Conversion (p) was calculated from the ^{1}H NMR spectrum (**Figure S7**) as

$$p = \frac{\int \mathrm{H}_{q'}}{\int \mathrm{H}_q + \int \mathrm{H}_{q'}} = \frac{\int \mathrm{H}_{4.60-5.16\ \mathrm{ppm}}}{\int \mathrm{H}_{4.60-5.30\ \mathrm{ppm}}}. \tag{S5}$$

Theoretical number-average molar masses ($M_{\mathrm{n,th}}$) of the graft copolymers, pLMA-*g*-pBzMA, were calculated as

$$M_{\mathrm{n,th}} = M_{\mathrm{mc,th}} + n_{\mathrm{CTA}}\mathrm{DP}_p M_{\mathrm{BzMA}} \tag{S6}$$

where $M_{\mathrm{mc,th}}$ is the theoretical molar mass of the functionalised main chain, $pLMA_{DP,tot}$-$CTA n_{CTA\%}$ (**Table S2**), and M_{BzMA} is the molar mass of BzMA.

Reinitiation efficiency of the CPADTC units (I_{init}) was calculated as

$$\mathrm{I}_{\mathrm{eff}} = \frac{\int \mathrm{H}_{H'}}{\int \mathrm{H}_H + \int \mathrm{H}_{H'}} = \frac{\int \mathrm{H}_{3.07-3.25\ \mathrm{ppm}}}{\int \mathrm{H}_{3.07-3.42\ \mathrm{ppm}}}. \tag{S7}$$

The apparent number of grafts (n_g) was calculated by taking into account the maximum number of grafting points (**Eq S2**) and the reinitiation efficiency of CPADTC as given by ^{1}H NMR (**Eq S7**). The apparent number of grafts was calculated as

$$n_{\mathrm{g}} = n_{\mathrm{CTA}} \cdot \mathrm{I}_{\mathrm{eff}} \,. \tag{S8}$$

The apparent grafting density was defined as

$$n_{\mathrm{g},\%} = \frac{n_{\mathrm{g}}}{\mathrm{DP}_{\mathrm{tot}}}, \tag{S9}$$

where DP_{tot} is the length of the main chain. The resulting apparent DP (DP_{app}) of the pBzMA grafts was calculated by correcting for the apparent number of grafts as

$$\mathrm{DP}_{\mathrm{app}} = \frac{n_{\mathrm{CTA}}\,\mathrm{DP}_p}{\mathrm{I}_{\mathrm{eff}}}, \tag{S10}$$

where DP_p is the graft length given by conversion assuming 100% reinitiation efficiency, that is

$$\mathrm{DP}_p = \frac{[\mathrm{BzMA}]_0}{n_{\mathrm{CTA}}}\, p_{\mathrm{BzMA}} \,. \tag{S11}$$

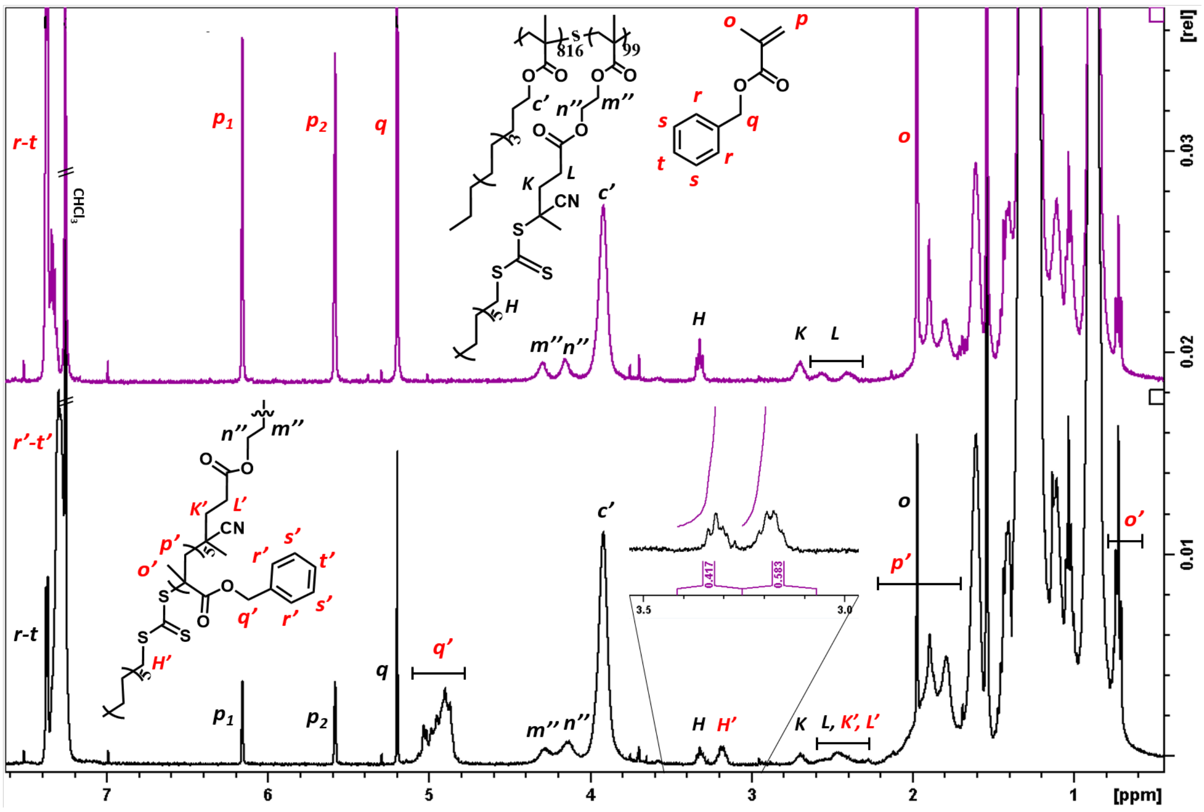

Figure S7 Representative ^{1}H NRM spectra (400 MHz, $CDCl_3$) of a dispersion polymerisation of BzMA before and after reaction using functionalised main chain $pLMA_{915}$-$CTA_{10\%}$ (**6**) and targeting graft length of 5 repeating units. 42% of CPADTC units remain unfragmented after reaction as indicated by shift δ = 3.32 → 3.19 ppm

3.4.1 *Effect of Graft Length*

The effect of graft length on the resulting morphologies was studied at 20 wt% by preparing a series of graft lengths using one main chain (**Table S3**). The appearances of the reaction mixtures after reaction are shown in **Figure S8**. SEC was carried out in $CHCl_3$ to confirm an increase in molecular weight with increasing graft length. The resulting materials **(6.1-6.12)** were characterised using SAXS without modification. SEM and TEM were used to visualise the nanostructures (**Figures S11 and S20**).

Table S3 Graft copolymers pLMA$_{915}$-*g*-pBzMA$_x$ prepared for studying the effect of graft length on PISA transitions using main chain pLMA$_{915}$-CTA$_{9\%}$.

Entry	Conversion ^{A} (%)	DP_p ^{B}	I_{eff} ^{C} (%)	DP_{app} ^{D}	$n_{g,\%}$ ^{E}	n_{BzMA}/n_{LMA} ^{F}	$M_{n,th}$ ^{G} (g/mol)	$M_{n,SEC}$ ^{H} (g/mol)	$Đ$ ^{H}
**(6.1)**	76	1	30	3	3	0.086	266,000	206064	1.82
**(6.2)**	79	2	45	4	4	0.17	279,000	203102	1.71
**(6.3)**	82	3	53	5	5	0.28	293,000	199158	1.76
**(6.4)**	84	4	57	6	5	0.36	305,000	217958	1.76
**(6.5)**	84	5	60	8	5	0.47	321,000	220816	1.73
**(6.6)**	91	10	69	14	6	1.0	394,000	236317	1.72
**(6.7)**	96	15	73	21	7	1.6	477,000	288389	1.56
**(6.8)**	99	18	74	24	7	1.8	518,000	315229	1.95
**(6.9)**	97	24	77	31	7	2.4	598,000	341057	1.68
**(6.10)**	99	31	78	40	7	3.1	696,000	382922	1.72
**(6.11)**	98	53	N/A	-	-	5.3	1,018,000	481065	1.50
**(6.12)**	99	105	N/A	-	-	11	1,773,000	660764	1.48

^{A} Determined by ^{1}H NMR (400 MHz, $CDCl_3$) (**Eq S5**). ^{B} Graft length as given by conversion assuming full reinitiation of CPADTC units (**Eq S11**). ^{C} Reinitiation efficiency of CPADTC units as given by ^{1}H NMR of end conversion sample. ^{D,E} Apparent graft length and grafting density, taking into account the reinitiation efficiency (**Eq S10** and **S9**). ^{F} Molar ratio of BzMA and LMA in the graft copolymer. ^{G} Theoretical number-average molar mass as given by conversion (**Eq S6**). ^{H} Experimental number-average molar mass and dispersity as given by SEC analysis in $CHCl_3$ with DRI detection and PMMA calibration.

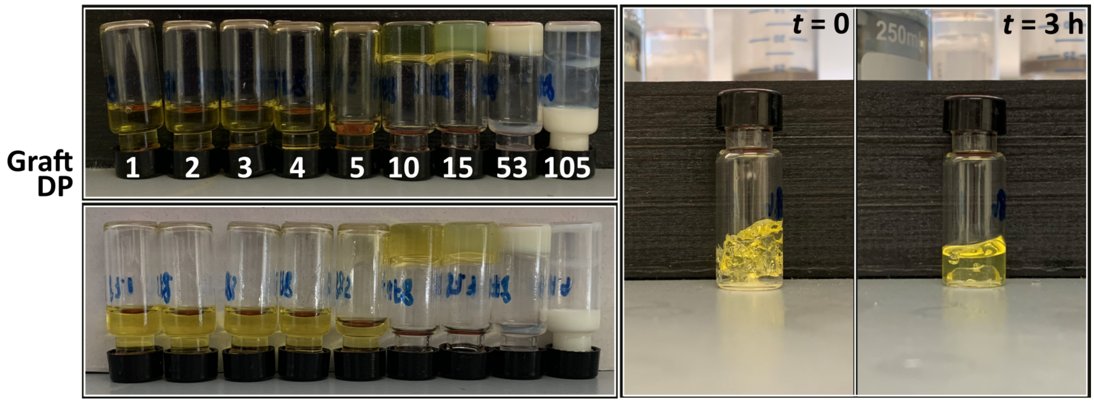

Figure S8 Left: Appearance of the reaction mixtures at room temperature after the PISA of pLMA$_{915}$-*g*-pBzMA$_x$, derived from main chain pLMA$_{915}$-CTA$_{10\%}$ at 20 wt%. Right: Recovery of gel-like material (**6.6**) after agitation.

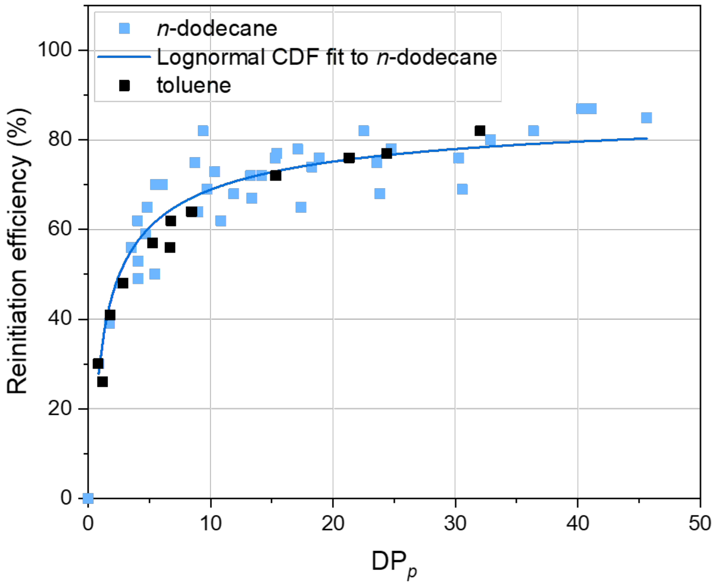

Figure S9 Graft length dependency of reinitiation efficiency across 44 PISA reactions in *n*-dodecane. Lognormal cumulative distribution fit was made into the data to calculate apparent graft lengths in the graft length series. A series of reactions was carried out in toluene as a control experiment. y0 = -158.7, A = 246.3, xc = -2.479, w = 3.335 and r^2 = 0.771.

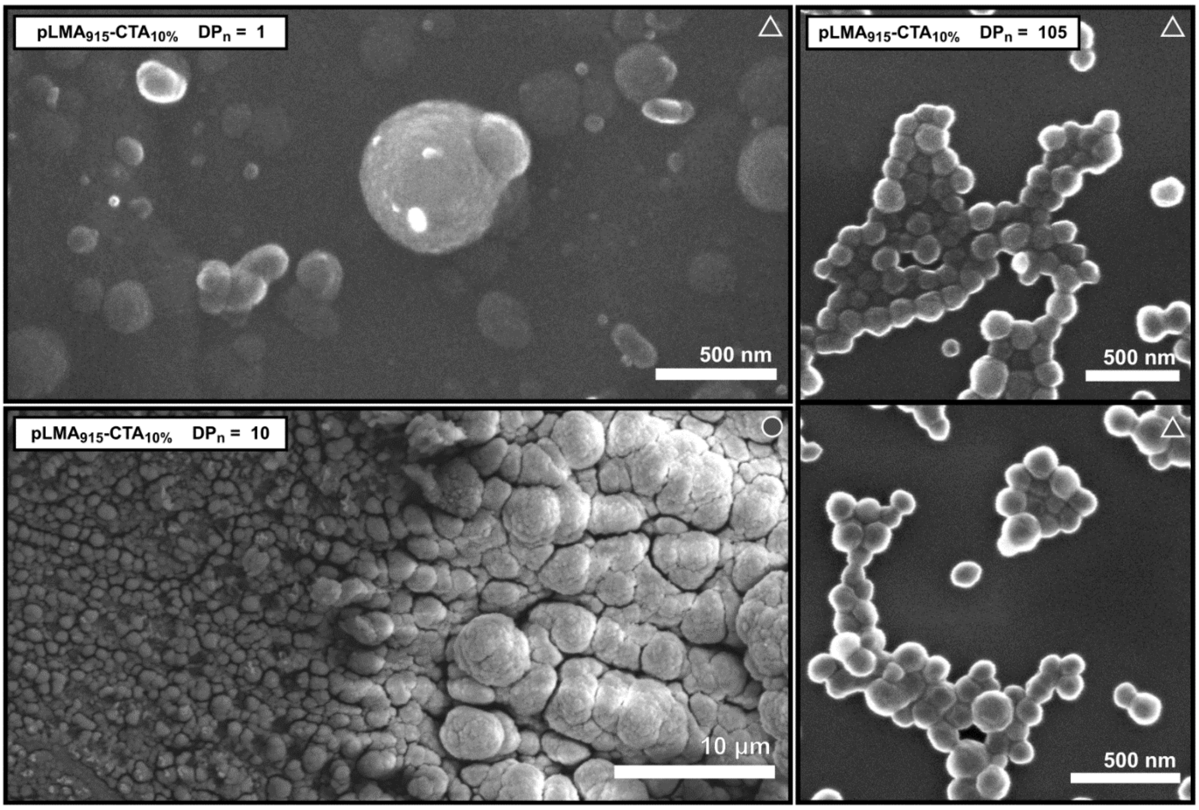

Figure S10 Supplementary SEM (▲) and cryo-SEM (●) images of nanostructures synthesised using $pLMA_{915}$-$CTA_{10\%}$ at 20 wt% solids.

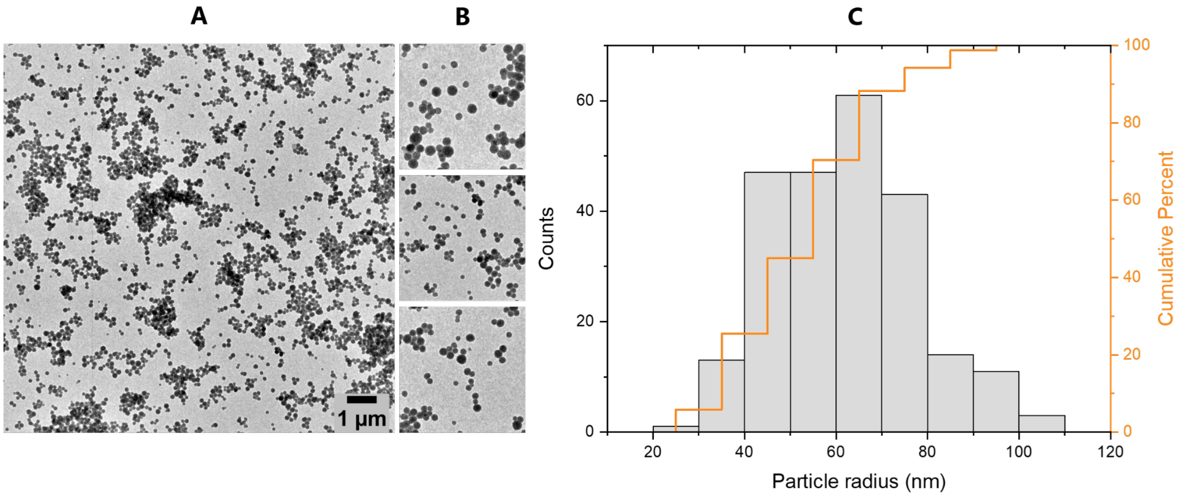

Figure S11 Particle size analysis of $pLMA_{915}$-*g*-$pBzMA_{105}$. **A**) Original TEM image. **B**) Three areas selected for analysis. **C**) Partizle size distribution as given by 240 manual measurements. Arithmetic mean = 62 nm.

Table S4 Parameters obtained through fitting SAXS data for samples of increasing graft length to a model consisting of a spherical form factor and a sticky hard sphere structure factor. Values marked with * were held constant throughout the fitting procedure.

	DP 1	DP 2	DP 3	DP 4	DP 5	DP 10	DP 15
**Solvent SLD ($\times 10^{-6}$ Å^{-2})**	7.41*	7.41*	7.41*	7.41*	7.41*	7.41*	7.41*
**Sphere SLD ($\times 10^{-6}$ Å^{-2})**	9.52*	9.52*	9.52*	9.52*	9.52*	9.52*	9.52*
**Volume fraction**	0.09 ± 0.0005	0.12 ± 0.0006	0.13 ± 0.0006	0.11 ± 0.0005	0.12 ± 0.0003	0.26 ± 0.0005	0.19 ± 0.0004
**Sphere radius (Å)**	16.0 ± 0.08	15.8 ± 0.07	16.4 ± 0.06	16.8 ± 0.09	17.9 ± 0.19	64.0 ± 0.05	67.2 ± 0.12
**Radial polydispersity**	0.16 ± 0.004	0.21 ± 0.003	0.21 ± 0.003	0.26 ± 0.002	0.35 ± 0.003	0.45 ± 0.002	0.40 ± 0.001
**Perturbation**	0.05*	0.05*	0.05*	0.05*	0.05*	0.05*	0.05*
**Stickiness**	0.22 ± 0.0014	0.21 ± 0.0008	0.16 ± 0.0003	0.13 ± 0.0021	0.12 ± 0.0004	0.44 ± 0.0004	0.59 ± 0.0063

Table S5 Parameters obtained through fitting SAXS data for increasing graft length from DP 18 to DP 37 to models consisting of a cylinder (DP 18) or flexible cylinder (DP 24, DP 37) form factor. Values marked with * were held constant throughout the fitting procedure.

	DP 18	DP 24	DP 37
**Solvent SLD (×10^{-6} Å^{-2})**	7.41*	7.41*	7.41*
**Shell SLD (×10^{-6} Å^{-2})**	9.52*	9.52*	9.52*
**Volume fraction**	0.25 ± 0.0001	0.14 ± 0.0001	0.12 ± 0.0004
**Cylinder length (Å)**	2000*	2000*	5000*
**Kuhn length (Å)**	-	107.1 ± 0.2	125.3 ± 1.3
**Cylinder radius (Å)**	48.0 ± 0.03	54.6 ± 0.02	59.4 ± 0.12
**Radial polydispersity**	0.32 ± 0.001	0.25 ± 0.001	0.35 ± 0.001

Table S6 Parameters obtained through fitting SAXS data for graft length DP 53 to a model consisting of a vesicle form factor. Values marked with * were held constant throughout the fitting procedure.

	DP 53
**Solvent SLD (×10^{-6} Å^{-2})**	7.41*
**Shell SLD (×10^{-6} Å^{-2})**	9.52*
**Volume fraction**	0.16 ± 0.00003
**Core radius (Å)**	173.9 ± 0.04
**Wall thickness (Å)**	92.3 ± 0.04
**Wall thickness polydispersity**	0.26 ± 0.001
**Radial polydispersity**	0.60 ± 0.001

Table S7 Parameters obtained through fitting SAXS data for graft length DP 100 to a raspberry form factor describing small pLMA spheres within a larger pBzMA particle. Values marked with * were held constant throughout the fitting procedure.

	DP 100
**Solvent SLD (×10^{-6} Å^{-2})**	7.41*
**Large particle SLD (×10^{-6} Å^{-2})**	9.52*
**Small particle SLD (×10^{-6} Å^{-2})**	8.22*
**Volume fraction, large**	0.25 ± 0.0003
**Volume fraction, small**	0.21 ± 0.0003
**Surface fraction**	0.25 ± 0.0018
**Radius, large (Å)**	569.5 ± 0.23
**Radius, small (Å)**	100.0 ± 0.06
**Fractional penetration**	1*
**Radial polydispersity**	0.25 ± 0.0002

3.4.2 *Effect of Main Chain Length*

The effect of main chain length on the PISA was studied at 20 wt% by polymerising a series of graft lengths with three functionalised main chains of dissimilar lengths but similar degrees of functionalisation (**Table S8**). The appearances of the reaction mixtures after reaction are shown in **Figure S12**. SEC was carried out to confirm an increase in molecular weight with increasing graft length (**Figure S13**). TEM was used to visualise some of the resulting materials.

Table S8 Graft copolymers pLMA$_x$-*g*-pBzMA$_y$ prepared for studying the effect of main chain length on PISA transitions using main chains of dissimilar lengths but similar degrees of functionalisation.

Main chain	Entry	Conversion [A] (%)	DP$_p$ [B]	n_{BzMA}/ n_{LMA} [C]	$M_{n,th}$ [D] (g/mol)	$M_{n,SEC}$ [E] (g/mol)	Đ [E]
pLMA$_{915}$-CTA$_{9\%}$	**(6.13)**	97	6	0.61	342,000	242,000	1.63
	**(6.14)**	97	12	1.2	426,000	282,000	1.66
	**(6.8)**	99	18	1.8	518,000	301,000	1.61
	**(6.9)**	97	24	2.4	598,000	333,000	1.63
	**(6.10)**	99	31	3.1	696,000	383,000	1.72
pLMA$_{474}$-CTA$_{10\%}$	**(7.1)**	86	5	0.49	170,000	128,000	1.27
	**(7.2)**	92	10	1.1	212,000	147,000	1.31
	**(7.3)**	97	15	1.6	256,000	158,000	1.32
	**(7.4)**	93	19	2.1	291,000	169,000	1.35
	**(7.5)**	98	26	2.8	341,000	200,000	1.31
pLMA$_{206}$-CTA$_{10\%}$	**(8.1)**	93	5	0.51	73,500	60,400	1.27
	**(8.2)**	97	9	1.0	89,000	72,000	1.27
	**(8.3)**	98	14	1.5	105,000	82,100	1.27
	**(8.4)**	97	19	2.0	121,000	91,900	1.27
	**(8.5)**	98	24	2.5	139,000	101,000	1.29

[A] Determined by ^{1}H NMR (400 MHz, $CDCl_3$) (**Eq S5**). [B] Graft length as given by conversion assuming full reinitiation of CPADTC units (**Eq S11**). [C] Molar ratio of BzMA and LMA in the graft copolymer. [D] Theoretical number-average molar mass as given by conversion (**Eq S6**). [E] Experimental number-average molar mass and dispersity as given by SEC analysis in $CHCl_3$ with DRI detection and PMMA calibration.

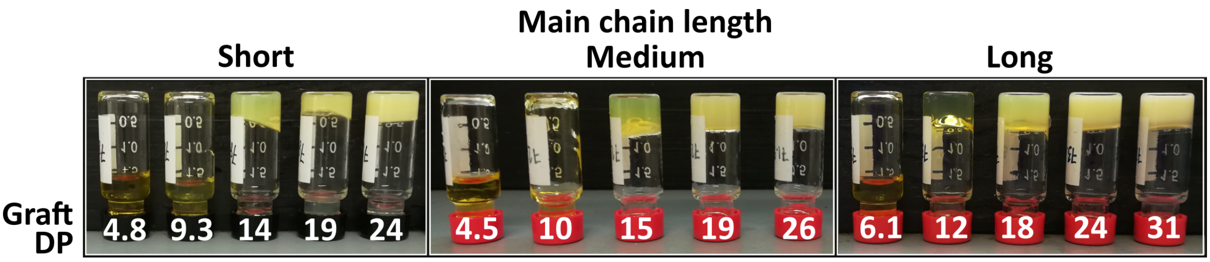

Figure S12 Appearance of the reaction mixtures of graft copolymers pLMA$_x$-*g*-pBzMA$_y$ at room temperature after the PISA using main chains (**6**-**8**) at 20 wt%. Left: Short main chain pLMA$_{206}$-CTA$_{10\%}$ (**8.1**-**8.5**). Middle: Medium-length main chain pLMA$_{474}$-CTA$_{10\%}$ (**7.1**-**7.5**). Right: Long main chain pLMA$_{915}$-CTA$_{10\%}$ (**6.8-6.10, 6.13 and 6.14**).

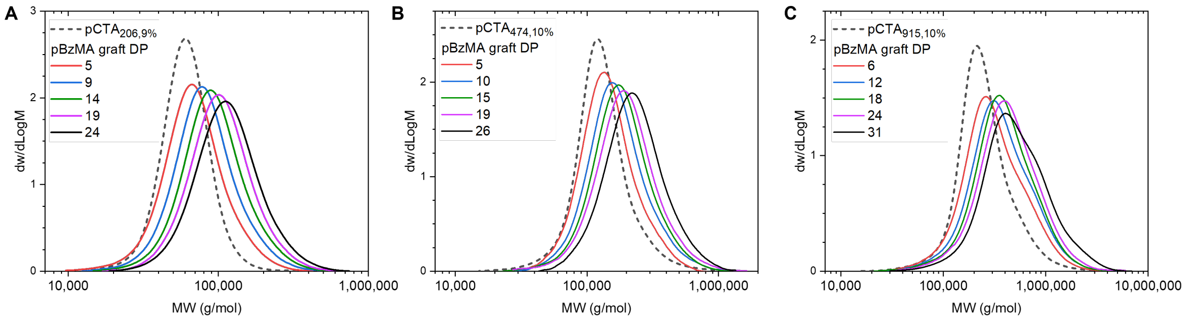

Figure S13 Size exclusion chromatograms of pLMA$_x$-*g*-pBzMA$_y$ graft copolymers prepared in the main chain length dependence study. Analysis was carried out using $CHCl_3$ as the eluent, DRI detection and PMMA calibration. A) Short main chain pLMA$_{206}$-CTA$_{10\%}$ (**8.1**-**8.5**). B) Medium-length main chain pLMA$_{474}$-CTA$_{10\%}$ (**7.1**-**7.5**). C) Long main chain pLMA$_{915}$-CTA$_{10\%}$ (**6.8-10, 6.13-14**).

3.4.3 Effect of Grafting Density

The effect of grafting density on the PISA was studied at 20 wt% by polymerising a series of graft lengths with three functionalised main chains with dissimilar degrees of functionalisation but similar lengths (**Table S9**). The appearances of the reaction mixtures after reaction are shown in (**Figure S14**). SEC was carried out to confirm an increase in molecular weight with increasing graft length (**Figure S15**).

Table S9 Graft copolymers pLMA$_x$-*g*-pBzMA$_y$ prepared for studying the effect of grafting density on PISA transitions using main chains with dissimilar degrees of functionalisation but similar lengths.

Main chain	Entry	Conversion [A] (%)	DP_p [B]	n_{BzMA}/n_{LMA} [C]	$M_{n,th}$ [D] (g/mol)	$M_{n,SEC}$ [E] (g/mol)	$Đ$ [E]
pLMA$_{915}$-CTA$_{10\%}$	**(6.10)**	97	6.1	0.61	342,000	242,000	1.63
	**(6.11)**	97	12	1.2	426,000	282,000	1.66
	**(6.12)**	99	18	1.8	518,000	301,000	1.61
	**(6.13)**	97	24	2.4	598,000	333,000	1.63
	**(6.14)**	99	31	3.1	696,000	383,000	1.72
pLMA$_{896}$-CTA$_{5\%}$	**(9.1)**	86	5.5	0.23	271,000	187,000	1.93
	**(9.2)**	89	10	0.44	301,000	204,000	1.81
	**(9.3)**	89	15	0.65	332,000	210,000	1.59
	**(9.4)**	98	23	0.95	378,000	258,000	1.65
	**(9.5)**	98	30	1.3	427,000	282,000	1.61
	**(9.6)**	99	36	1.5	465,000	277,000	1.60
pLMA$_{939}$-CTA$_{2\%}$	**(10.1)**	83	4	0.076	255,000	155,000	1.74
	**(10.2)**	89	9	0.16	269,000	164,000	1.69
	**(10.3)**	92	13	0.25	283,000	169,000	1.64
	**(10.4)**	96	19	0.34	297,000	182,000	1.45
	**(10.5)**	97	92	1.71	519,000	224,000	2.41

[A] Determined by ^{1}H NMR (400 MHz, $CDCl_3$) (**Eq S5**). [B] Graft length as given by conversion assuming full reinitiation of CPADTC units (**Eq S11**). [C] Molar ratio of BzMA and LMA in the graft copolymer. [D] Theoretical number-average molar mass as given by conversion (**Eq S6**). [E] Experimental number-average molar mass and dispersity as given by SEC analysis in $CHCl_3$ with DRI detection and PMMA calibration.

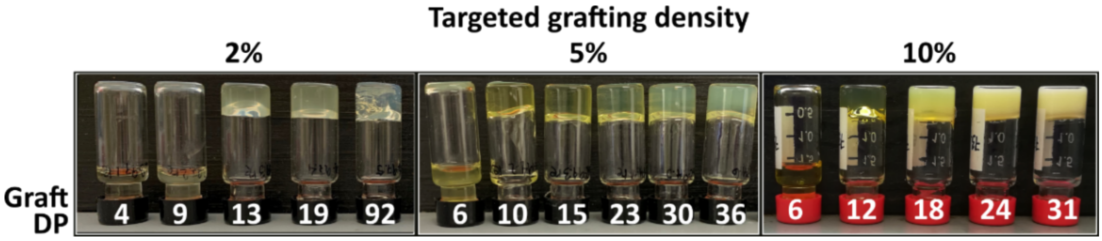

Figure S14 Appearance of the reaction mixtures of graft copolymers pLMA$_x$-*g*-pBzMA$_y$ at room temperature after the PISA using main chains with different degrees of functionalisation at 20 wt%. Left: Main chain pLMA$_{939}$-CTA$_{2\%}$ (**10.1-10.5**). Middle: Main chain pLMA$_{896}$-CTA$_{4\%}$ (**9.1-9.6**). Right: Main chain pLMA$_{915}$-CTA$_{9\%}$ (**6.10-6.14**).

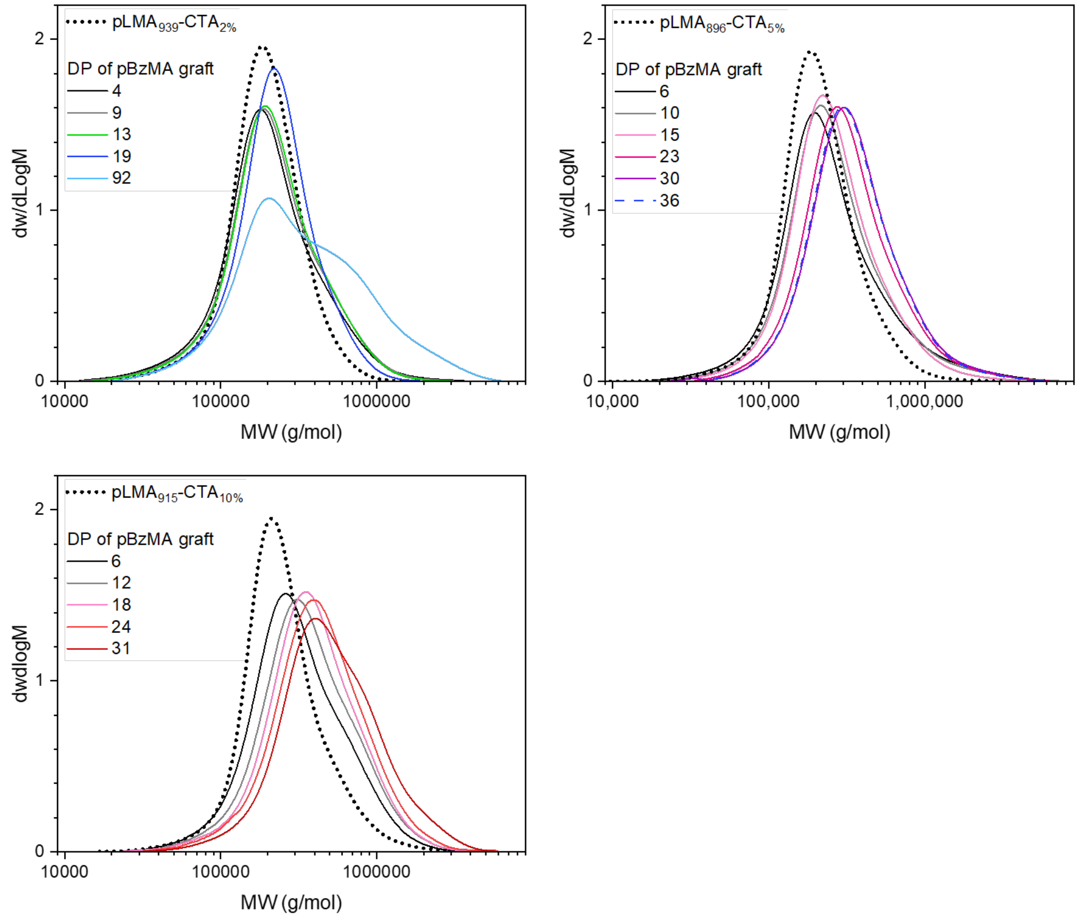

Figure S15 SEC profiles of pLMA-*g*-pBzMA graft copolymers prepared targeting various graft lengths and grafting densities**.** Analysis was conducted in $CHCl_3$ with DRI detection and PMMA calibration.

3.4.4 *Effect of Concentration*

The effect of total mass concentration on the PISA was studied at 10 wt% and 20 wt% by polymerising two series of graft lengths with each of three functionalised main chains of dissimilar lengths but similar degrees of functionalisation (**Table S10**). The appearances of the reaction mixtures after reaction are shown in (**Figure S16**). SEC was carried out to confirm an increase in molecular weight with increasing graft length (**Figure S17**).

Table S10 Graft copolymers $pLMA_x$-*g*-$pBzMA_y$ prepared for studying the effect of total mass concentration on PISA transitions using main chains of dissimilar lengths but similar degrees of functionalisation.

Main chain	Concentration (wt%)	Entry	Conversion [A] (%)	DP_p [B]	n_{BzMA}/n_{LMA} [C]	$M_{n,th}$ [D] (g/mol)	$M_{n,SEC}$ [E] (g/mol)	*Đ* [E]
		**(6.15)**	88	6	0.61	333,000	220,000	1.77
		**(6.16)**	87	11	1.2	411,000	291,000	1.54
	**10**	**(6.17)**	91	17	1.9	501,000	307,000	1.68
		**(6.18)**	96	24	2.7	595,000	365,000	1.60
**$pLMA_{915}$-$CTA_{10\%}$**		**(6.19)**	96	30	3.4	689,000	391,000	1.53
		**(6.10)**	97	6	0.68	354,000	242,000	1.63
		**(6.11)**	97	12	1.3	448,000	282,000	1.66
	**20**	**(6.12)**	99	18	2.1	552,000	301,000	1.61
		**(6.13)**	97	24	2.7	641,000	333,000	1.63
		**(6.14)**	99	31	3.4	750,000	383,000	1.72
		**(7.7)**	86	9	1.0	206,000	134,000	1.29
		**(7.8)**	85	13	1.5	241,000	155,000	1.28
		**(7.9)**	80	17	1.9	274,000	165,000	1.31
	**10**	**(7.10)**	N/A [F]	(27 [G])	-	-	187,400 [F]	1.36
**$pLMA_{474}$-$CTA_{10\%}$**		**(7.11)**	N/A [F]	(42 [G])	-	-	230,000 [F]	1.40
		**(7.1)**	86	5	0.49	170,000	128,000	1.27
		**(7.2)**	92	10	1.1	212,000	147,000	1.31
	**20**	**(7.4)**	93	19	2.1	291,000	169,000	1.35
		**(7.5)**	98	26	2.8	341,000	200,000	1.31
		**(7.6)**	97	41	4.4	463,000	235,000	1.35
		**(8.6)**	83	4	0.54	70,900	58,300	1.26
		**(8.7)**	89	9	1.2	86,800	68,100	1.28
		**(8.8)**	N/A [F]	(20 [G])	-	-	-	-
	**10**	**(8.9)**	N/A [F]	(24 [G])	-	-	148,000 [F]	1.46
**$pLMA_{206}$-$CTA_{11\%}$**		**(8.10)**	N/A [F]	(48 [G])	-	-	-	-
		**(8.1)**	93	5	0.51	73,500	60,400	1.27
		**(8.2)**	97	9	1.0	89,000	72,000	1.27
	**20**	**(8.3)**	98	14	1.5	105,000	82,100	1.27
		**(8.4)**	97	19	2.0	121,000	91,900	1.27
		**(8.6)**	98	47	5.0	218,000	156,000	1.28

[A] Determined by ^{1}H NMR (400 MHz, $CDCl_3$) (**Eq S5**). [B] Graft length as given by conversion assuming full reinitiation of CPADTC units (**Eq S11**). [C] Molar ratio of BzMA and LMA in the graft copolymer. [D] Theoretical number-average molar mass as given by conversion (**Eq S6**). [E] Experimental number-average molar mass and dispersity as given by SEC analysis in $CHCl_3$ with DRI detection and PMMA calibration. [F] Sampling unreliable due to heterogeneity of reaction mixture. [G] Targeted graft length.

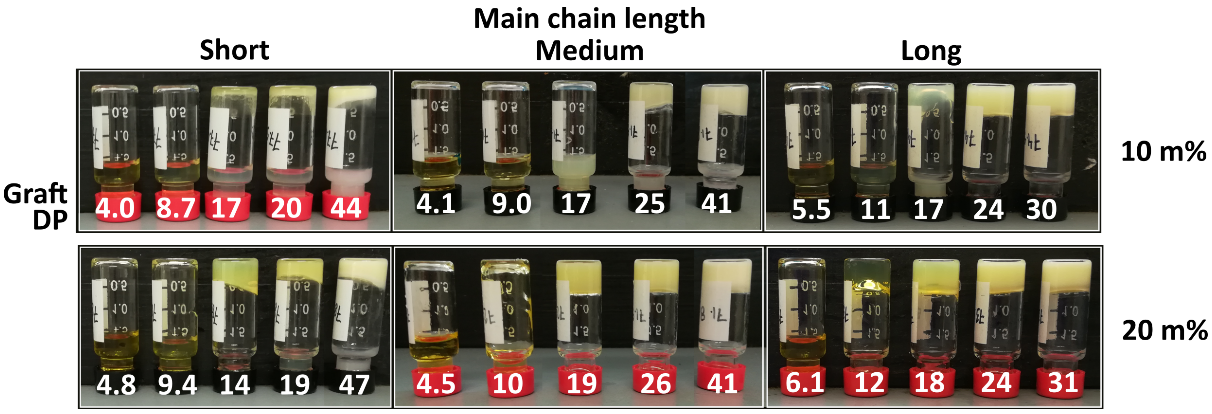

Figure S16 **Appearance of the reaction mixtures of graft copolymers pLMA$_x$-*g*-pBzMA$_y$ at room temperature after the PISA using main chains (6-8) at 10 wt% and 20 wt%. Left: Short main chain** pLMA$_{206}$-CTA$_{10\%}$ **reactions (**8.1-8.10**). Middle: Medium-length main chain** pLMA$_{474}$-CTA$_{10\%}$ **reactions (**7.1-7.10**). Right: Long main chain** pLMA$_{915}$-CTA$_{10\%}$ **reactions (**6.10-6.19**).**

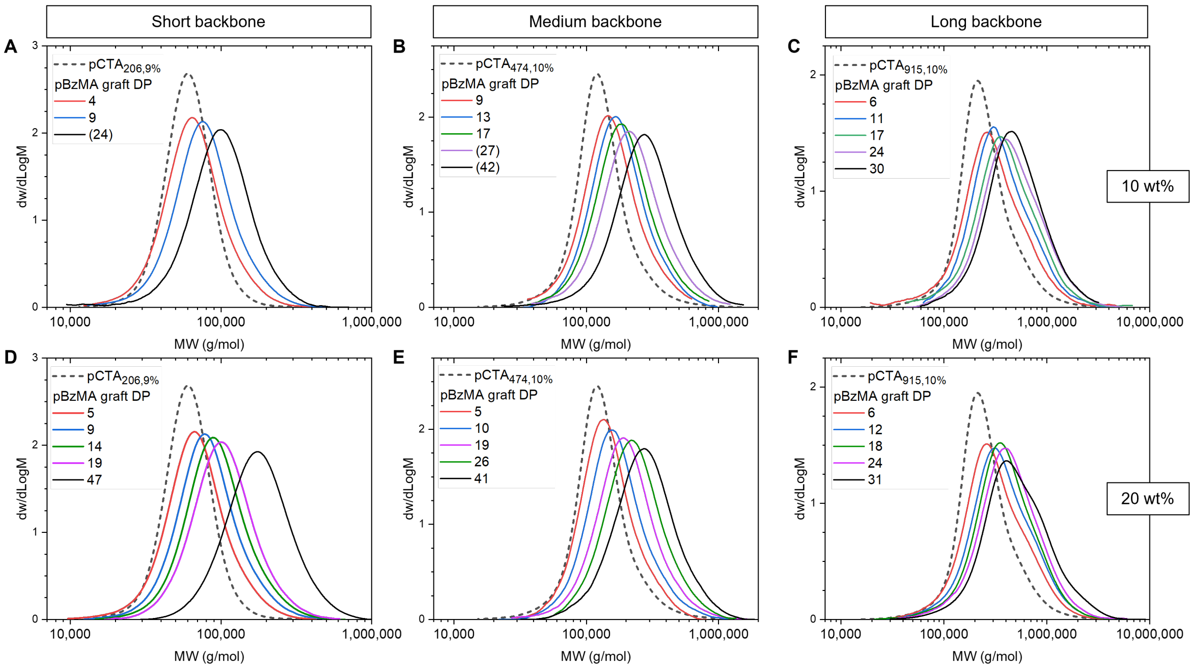

Figure S17 Size exclusion chromatograms of pLMA$_x$-*g*-pBzMA$_y$ graft copolymers prepared at 10 wt% (A-C) and 20 wt% (D-F) in the concentration dependence study. Analysis was carried out using $CHCl_3$ as the eluent, DRI detection and PMMA calibration. Graft lengths indicated in brackets could not be reliably quantified with ^{1}H NMR and correspond to targeted DP.

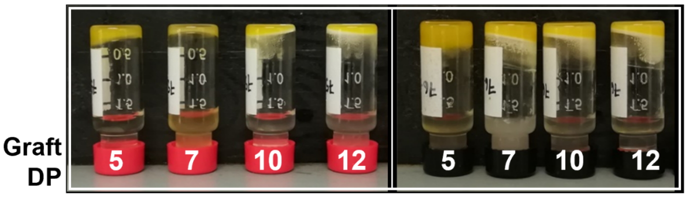

Figure S18 Appearances of reaction mixtures after PISA using $pLMA_{194}$-$CTA_{29\%}$ to target graft DPs 5-12 at 10 wt% (left) and 20 wt% (right).

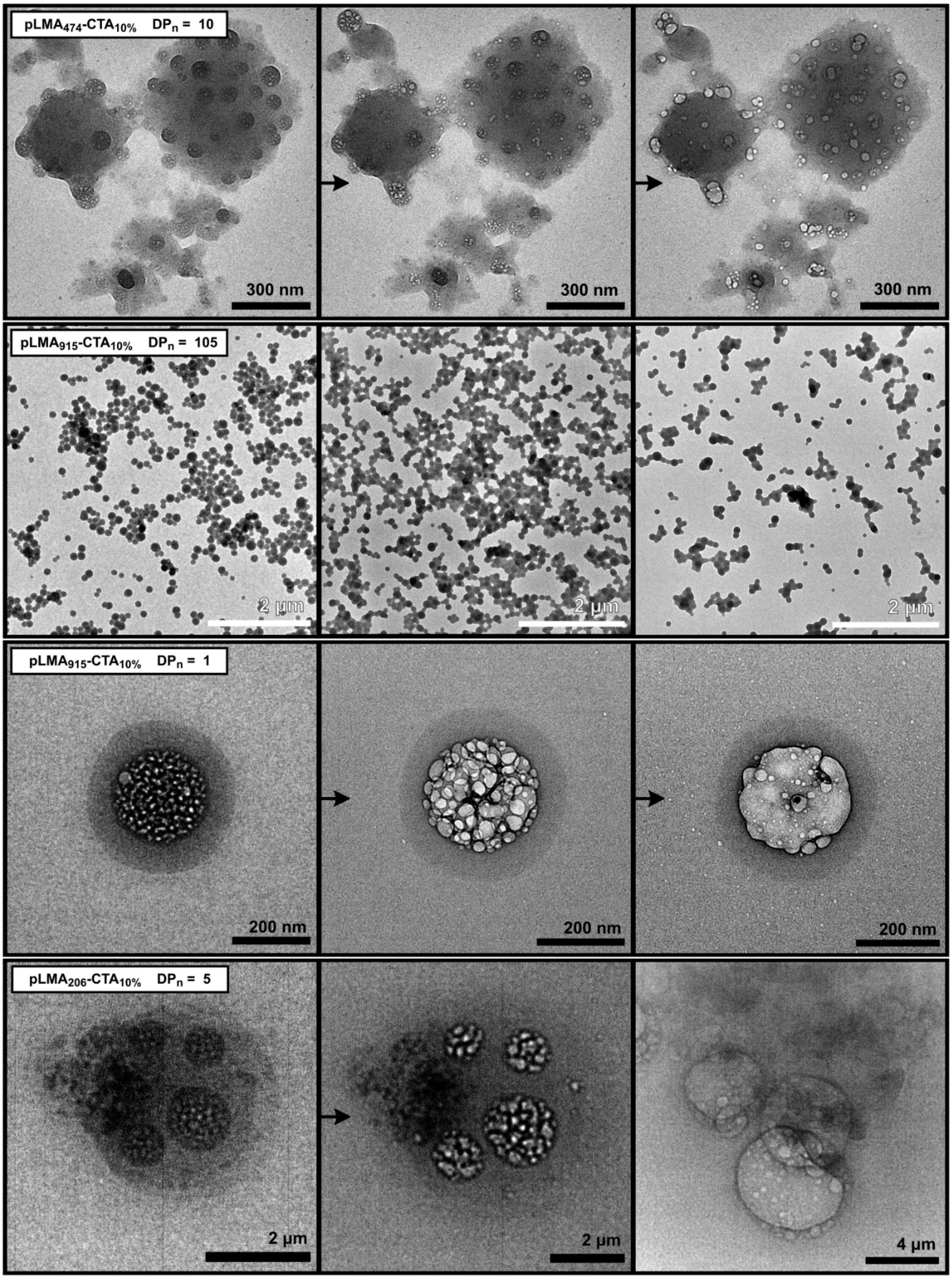

Figure S19 Destructive effects of the electron beam. Images show loss of colloidal shape and internal morphology of nanostructures prepared at 20 wt% solids.

4 References

1. David, G.; Pérez, J., Combined Sampler Robot and High-Performance Liquid Chromatography: A Fully Automated System for Biological Small-Angle X-Ray Scattering Experiments at the Synchrotron SOLEIL SWING Beamline. *J. Appl. Crystallogr.* **2009,** *42* (5), 892-900.
2. Zhang, F.; Ilavsky, J.; Long, G. G.; Quintana, J. P. G.; Allen, A. J.; Jemian, P. R., Glassy Carbon as an Absolute Intensity Calibration Standard for Small-Angle Scattering. *Metallurgical and Materials Transactions A* **2010,** *41* (5), 1151-1158.
3. SasView. https://www.sasview.org/.
4. Clifton, L. A.; Hall, S. C. L.; Mahmoudi, N.; Knowles, T. J.; Heinrich, F.; Lakey, J. H., *Structural Investigations of Protein–Lipid Complexes Using Neutron Scattering*. Springer New York: New York, 2019.
5. Menon, S. V. G.; Manohar, C.; Rao, K. S., A new interpretation of the sticky hard sphere model. *The Journal of Chemical Physics* **1991,** *95* (12), 9186-9190.
6. Guinier, A.; Fournet, G., *Small-Angle Scattering of X-Rays*. Wiley: 1955.
7. Pedersen, J. S., Analysis of small-angle scattering data from colloids and polymer solutions: modeling and least-squares fitting. *Advances in Colloid and Interface Science* **1997,** *70*, 171-210.
8. Pedersen, J. S.; Schurtenberger, P., Scattering Functions of Semiflexible Polymers with and without Excluded Volume Effects. *Macromolecules* **1996,** *29* (23), 7602-7612.
9. Chen, W.-R.; Butler, P. D.; Magid, L. J., Incorporating Intermicellar Interactions in the Fitting of SANS Data from Cationic Wormlike Micelles. *Langmuir* **2006,** *22* (15), 6539-6548.
10. Larson-Smith, K.; Jackson, A.; Pozzo, D. C., Small angle scattering model for Pickering emulsions and raspberry particles. *Journal of Colloid and Interface Science* **2010,** *343* (1), 36-41.
11. Debye, P., Molecular-weight Determination by Light Scattering. *The Journal of Physical and Colloid Chemistry* **1947,** *51* (1), 18-32.
12. Feigin, L. A.; Svergun, D. I., *Structure Analysis by Small-Angle X-Ray and Neutron Scattering*. Springer: 1987.
13. Zhang, Q.; Hong, J.-D.; Hoogenboom, R., A triple thermoresponsive schizophrenic diblock copolymer. *Polymer Chemistry* **2013,** *4* (16), 4322-4325.
14. Moad, G.; Chong, Y. K.; Postma, A.; Rizzardo, E.; Thang, S. H., Advances in RAFT polymerization: the synthesis of polymers with defined end-groups. *Polymer* **2005,** *46* (19), 8458-8468.
